# Cannabis and tobacco co-use predicts psychosis in clinical high risk cohorts

**DOI:** 10.1038/s44220-026-00648-y

**Published:** 2026-05-12

**Authors:** Daniel Bello, Sophia H. Blyth, Rachel A. Rabin, Jean Addington, Carrie E. Bearden, Kristin Cadenhead, Tyrone D. Cannon, Ricardo E. Carrión, Barbara Cornblatt, Matcheri Keshavan, Daniel H. Mathalon, Diana O. Perkins, Larry Seidman, William S. Stone, Ming T. Tsuang, Elaine F. Walker, Scott Woods, Roscoe O. Brady, Heather Burrell Ward

**Affiliations:** 1 Department of Psychiatry and Behavioral Sciences, Vanderbilt University Medical Center, Nashville, TN USA; 2 Department of Psychiatry, McGill University, Montreal, Quebec Canada; 3 The Douglas Mental Health University Institute, Verdun, Quebec Canada; 4 Department of Psychiatry, Hotchkiss Brain Institute, University of Calgary, Calgary, Alberta Canada; 5 Semel Institute for Neuroscience and Human Behavior, Departments of Psychiatry and Behavioral Sciences and Psychology, University of California Los Angeles, Los Angeles, CA USA; 6 Department of Psychiatry, University of California San Diego, La Jolla, CA USA; 7 Department of Psychology and Psychiatry, Yale University, New Haven, CT USA; 8 Department of Psychiatry, Zucker Hillside Hospital and Donald and Barbara Zucker School of Medicine at Hofstra/Northwell, Glen Oaks, NY USA; 9 Department of Psychiatry, Beth Israel Deaconess Medical Center and Harvard Medical School, Boston, MA USA; 10 Department of Psychiatry and Behavioral Sciences, University of California San Francisco, San Francisco, CA USA; 11 Veterans Affairs San Francisco Health Care System, San Francisco, CA USA; 12 Department of Psychiatry, University of North Carolina at Chapel Hill, Chapel Hill, NC USA; 13 Department of Psychology, Emory University, Atlanta, GA USA; 14 Department of Psychiatry, Yale University, New Haven, CT USA

**Keywords:** Psychosis, Addiction

## Abstract

Cannabis and tobacco use are highly prevalent among people with psychosis and are associated with medical comorbidities and poor prognosis. Concurrent use of cannabis and tobacco (‘co-use’) is rising in the general population, but has not been studied in psychosis. Given the devastating consequences of cannabis and tobacco use, it is critical to understand how their co-use affects psychiatric symptoms and the development of psychosis. Here we used the North American Prodrome Longitudinal Study-2, a multisite prospective study of individuals at clinical high risk for psychosis (CHR), and healthy controls to examine baseline differences in psychiatric symptoms and conversion to psychosis across substance groups: (1) CHR tobacco use, (2) CHR cannabis use, (3) CHR co-use, (4) CHR non-tobacco or cannabis use, (5) CHR without substance use and (6) healthy controls. Among 1,012 participants (734 CHR, 278 controls), more frequent cannabis and tobacco use was linked to greater psychiatric symptom severity. In survival analyses, heavy cannabis and light tobacco co-use (HR = 2.93, 95% confidence interval (CI) [1.23–6.97], *P* = 0.015) was associated with higher risk of conversion than no use of either substance. Co-use of tobacco and cannabis was not associated with psychiatric symptom severity but did predict higher risk of conversion to psychosis. These results highlight the need for strategies that address co-use in CHR populations to mitigate potential long-term psychiatric consequences.

## Abstract (web summary)

In individuals at clinical high risk for psychosis, the concurrent use of cannabis and tobacco predicted an elevated risk of transitioning to psychosis. Heavy cannabis use combined with light tobacco use was linked to a threefold increase in the risk of transition.

## Main

Cannabis and tobacco use are highly prevalent among people with psychosis^1^. Up to 62% of people with schizophrenia use tobacco—a prevalence three times that of the general population^2^. Cannabis use is also common, especially among individuals with first episode psychosis (FEP)^3^. Across schizophrenia spectrum disorders, cannabis use disorders are present in 26.2% of individuals, with prevalence rising to 36% among those with FEP^4,5^.

Tobacco and cannabis use are associated with substantial medical and psychiatric comorbidities in people with psychosis. Tobacco use is the leading preventable cause of early mortality in schizophrenia, leading to a 20-year decreased life expectancy compared to the general population^2,6,7^. Individuals with psychosis smoke more heavily, are more nicotine-dependent, and are less likely to quit smoking than people without a psychotic disorder^2,8,9^.

Cannabis use is associated with psychotic symptom exacerbation, psychotic relapse, treatment nonadherence and poorer overall functioning^10–13^. Lifetime cannabis use is associated with a 1.4-fold increase in the risk of developing psychotic illness, with cannabis dependence conferring a 3.4-fold increase^10^. Higher frequency of cannabis use is also associated with higher psychosis risk, especially in adolescence^14,15^. Cannabis use is involved in roughly half of psychotic disorder cases, and regular use is a predictor of heightened schizophrenia risk^16–20^. Cannabis use is known to cause positive symptoms of psychosis, including paranoia and hallucinations^21,22^, as well as anhedonia and amotivation^23,24^.

Substance use generally begins in adolescence, prior to the onset of psychosis. Individuals who use tobacco are at increased risk of developing psychosis^25^, and individuals at clinical high risk for psychosis (CHR) are more likely to smoke cigarettes compared to controls^26,27^. Individuals who use cannabis have onset of schizophrenia two to three years earlier than those who do not use cannabis^10^.

Existing literature has studied the effects of single substances (that is, tobacco or cannabis use) on psychiatric symptoms and treatment outcomes in the psychosis prodrome. Tobacco and cannabis use are consistently reported at higher rates in the CHR population, although findings are mixed regarding their association with risk of conversion to psychosis^26–30^. However, the real world is not a controlled trial of tobacco or cannabis use alone, as many individuals use both substances.

Concurrent use of tobacco and cannabis, defined as ‘co-use’, has become increasingly prevalent in the general population. Co-use includes using substances at the same time, on the same occasion, or within a defined timeframe where their effects may overlap. Co-use of tobacco and cannabis can occur simultaneously, such as spliffs, which are joints that include cannabis and loose-leaf tobacco, or asynchronously, where the individual uses both substances at different times (for example, using tobacco in the morning and cannabis at night)^16^. In 2018, among individuals aged 18–25 years in the United States, 48% of tobacco users report cannabis use in the past month^31^. In 2021, US adolescents reported co-use at rates similar to tobacco use alone and higher than cannabis use alone^32^. Co-use of tobacco and cannabis is associated with higher rates of psychiatric conditions, worse substance use outcomes and poorer physical health^16,32^. Individuals in their first episode of psychosis who co-use tobacco and cannabis have an earlier age of onset and more severe symptom profile than those who use tobacco or cannabis alone^33,34^.

Given the devastating medical and psychiatric consequences of use of these substances, it is critical to understand the effects of co-use on psychiatric symptoms and risk of developing a psychotic disorder. Because substance use often begins before the onset of psychosis, we investigated tobacco and cannabis co-use during the CHR period, a time prior to the onset of psychosis when individuals experience attenuated psychotic symptoms, alterations in mood and anxiety, and changes in social functioning^35,36^. We compared baseline symptom severity among CHR with current co-use, tobacco use only or cannabis use only, other substance use, and no substance use from the North American Prodrome Longitudinal Study (NAPLS2), a multisite prospective study of individuals at risk for psychosis and healthy controls who underwent neuroimaging and clinical characterization then were followed for two years for development of a psychotic disorder (that is, conversion to psychosis). We also assessed whether baseline tobacco and cannabis co-use was associated with conversion to psychosis. We hypothesized that CHR with co-use would have (1) more severe psychiatric symptoms and (2) a higher rate of conversion to psychosis than CHR using only tobacco or cannabis.

## Results

There were 1,012 participants with data for analysis. Healthy controls (*n* = 278) were older than CHR individuals (19.75 versus 18.48 years, *t*(458.3) = 3.91, *P* < 0.001, *n* = 734). CHR substance use groups differed significantly in age (*F*(4,729) = 36.74, *P* < 0.001), with the Non-Substance Use group being younger than all other CHR groups (all Bonferroni-corrected pairwise *P* < 0.001; Table 1). Groups also differed in sex distribution (*χ*^2^(5) = 16.04, *P* = 0.007), with the co-use group being more male than the Non-Substance Use, Non-TC Use, and Healthy Control groups. There were no significant differences in race or socioeconomic status between CHR substance use groups (Table 1 and Supplementary Fig. 1). The Non-Substance Use group was more Hispanic than the Co-Use group (Fisher’s exact test, *P* = 0.002; Table 1).

**Table 1:** Demographics by substance use group

	Clinical high risk (*n* = 734)					Healthy Control (*n* = 278)
	Co-Use (*n* = 97)	Tobacco Use (*n* = 78)	Cannabis Use (*n* = 79)	No Tobacco or Cannabis Use (*n* = 105)	No Substance Use (*n* = 375)	
**Age, yr (s.d.)**^**a**^	19.3 (3.0)	20.3 (4.2)	19.6 (3.8)	21.2 (4.7)	16.9 (3.7)	19.7 (4.7)
**Sex, female (%)**^**b**^	28 (28.9)	29 (37.2)	33 (41.8)	53 (50.5)	168 (44.8)	138 (49.6)
**Sex, male (%)**^**c**^	69 (71.1)	49 (62.8)	46 (58.2)	52 (49.5)	207 (55.2)	140 (50.4)
**Race (%)**						
First Nations	2 (2.1)	0 (0.0)	1 (1.3)	0 (0.0)	10 (2.7)	4 (1.4)
East Asian	1 (1.0)	1 (1.3)	4 (5.1)	1 (1.0)	12 (3.2)	15 (5.4)
Southeast Asian	0 (0.0)	2 (2.6)	0 (0.0)	0 (0.0)	13 (3.5)	7 (2.5)
South Asian	1 (1.0)	2 (2.6)	1 (1.3)	2 (2.0)	13 (3.5)	8 (2.9)
Black	13 (13.4)	9 (11.5)	12 (15.2)	17 (16.2)	58 (15.5)	48 (17.3)
Central/South American	1 (1.0)	4 (5.1)	2 (2.5)	4 (3.8)	21 (5.6)	13 (4.7)
West/Central Asia and Middle East	1 (1.0)	2 (2.6)	0 (0.0)	0 (0.0)	3 (0.8)	2 (0.7)
White	70 (72.2)	49 (62.8)	52 (65.8)	64 (61.0)	191 (51.1)	151 (54.3)
Native Hawaiian or Pacific Islander	0 (0.0)	0 (0.0)	0 (0.0)	0 (0.0)	2 (0.5)	1 (0.4)
Interracial	8 (8.3)	9 (11.5)	7 (8.9)	17 (16.2)	51 (13.6)	29 (10.4)
Hispanic (%)^c^	9 (9.3)	8 (10.3)	12 (15.2)	22 (21.0)	87 (23.2)	49 (17.6)
**Taking antipsychotic (%)**	10.3	5.1	6.3	6.7	8.5	–
**CPZ equivalent dose**^**d**^	223.1 (144.2)	218.0 (210.1)	238.3 (174.9)	125.1 (114.3)	191.1 (150.1)	–
**SOPS (s.d.)**						
Positive	12.56 (3.9)	11.74 (4.1)	12.49 (4.1)	11.7 (3.6)	11.53 (3.7)	1.05^e^ (1.7)
Negative	11.62 (5.7)	11.78 (5.8)	12.0 (5.4)	11.62 (6.6)	12.05 (6.2)	1.44^e^ (2.2)
Disorganization	5.46 (3.1)	5.12 (2.9)	5.16 (3.1)	4.56 (3.3)	5.26 (3.2)	0.65^e^ (1.2)
General	9.99 (4.3)	9.49 (4.6)	9.34 (3.9)	9.29 (4.5)	8.82 (4.2)	1.30^e^ (2.1)
**SAS (s.d.)**	39.17 (10.7)	40.51 (11.4)	37.85 (11.3)	38.4 (11.0)	36.94 (10.5)	22.99^e^ (3.6)
**SIAS (s.d.)**	28.64 (17.2)	30.36 (16.2)	33.41 (16.2)	32.0 (17.0)	30.76 (18.0)	9.04^e^ (8.7)
**CDSS (s.d.)**	6.35 (5.2)	6.32 (4.7)	6.55 (4.8)	6.57 (5.0)	5.26 (4.5)	0.57^e^ (1.4)

CDSS, Calgary Depression Scale for Schizophrenia; CPZ, chlorpromazine equivalent; SAS, Self-Rating Anxiety Scale; SIAS, Social Interaction Anxiety Scale; SOPS, Scale of Psychosis-Risk Symptoms. Group differences were assessed using a two-sample Welch *t*-test for age, one-way analysis of variance (ANOVA) with Bonferroni-corrected pairwise *t*-tests for symptom severity, and Pearson *χ*^2^ or Fisher’s exact tests for categorical variables (sex, race, ethnicity and antipsychotic use). All tests were two-sided (*n* = 1,012). ^a^Age differed significantly across CHR substance use groups (*F*(4,729) = 36.74, *P* < 0.001); the Non-Substance Use group was younger than all other CHR groups (all Bonferroni-corrected pairwise *P* < 0.001). Healthy controls were also significantly older compared to all CHR individuals (*t*(458.3) = 3.91, *P* < 0.001). ^b^Co-Use group was more male than the Non-TC Use, Non-Substance Use and Healthy Control groups (*χ*^2^(5) = 16.04, *P* = 0.007; all Bonferroni-corrected pairwise *P* < 0.010). ^c^The Non-Substance Use group was more Hispanic than the Co-Use group (Fisher’s exact test,odds ratio = 2.96, 95% CI [1.41, 6.96], *P* = 0.002). ^d^If taking antipsychotic medication. ^e^The Healthy Control group had lower symptom severity on all scales compared to all CHR groups (*P* < 0.001).

### Individuals with co-use and single substance use have comparable frequency of use

There were no differences in cannabis use frequency between the Cannabis Use and Co-Use groups (*H*(1) = 0.77, *P* = 0.38; Supplementary Fig. 2a) nor in tobacco use frequency between the Tobacco Use and Co-Use groups (*H*(1) = 1.92, *P* = 0.17; Supplementary Fig. 2b). Alcohol use did not differ between the Tobacco Use, Cannabis Use and Co-Use groups (*H*(2) = 0.38, *P* = 0.83; Supplementary Fig. 3).

### Greater tobacco and cannabis use are associated with greater psychiatric symptom severity

**Fig. 1:**
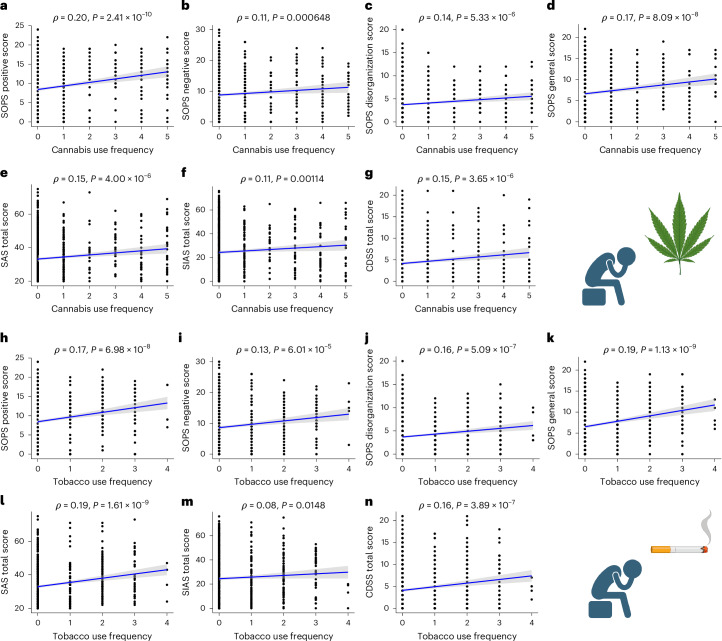
Cannabis and tobacco use frequency are associated with higher psychiatric symptom severity. **a**–**g**, Across the combined sample (CHR and Healthy Controls), more frequent cannabis use was associated with worsened psychiatric symptoms of psychosis across all SOPS domains (**a**, positive: *ρ* = 0.20, *P* < 0.001; **b**, negative: *ρ* = 0.11, *P* < 0.001; **c**, disorganization: *ρ* = 0.14, *P* < 0.001; **d**, general: *ρ* = 0.17, *P* < 0.001; **e**, anxiety: *ρ* = 0.15, *P* < 0.001; **f**, social anxiety: *ρ* = 0.11, *P* = 0.002; **g**, depression: *ρ* = 0.15, *P* < 0.001; Bonferroni-corrected *P* = 0.05/seven symptom domains = 0.007). **h**–**l**,**n**, Similarly, more frequent tobacco use was also associated with worsened psychiatric symptoms of psychosis (**h**, positive: *ρ* = 0.17, *P* < 0.001; **i**, negative: *ρ* = 0.13, *P* < 0.001; **j**, disorganization: *ρ* = 0.16, *P* < 0.001; **k**, general: *ρ* = 0.19, *P* < 0.001; **l**, anxiety: *ρ* = 0.19, *P* < 0.001; **n**, depression: *ρ* = 0.16, *P* < 0.001; Bonferroni-corrected *P* = 0.05/7 symptom domains = 0.007). Sample sizes varied across panels due to missing data: **a**, *n* = 1,010; **b**, *n* = 996; **c**, *n* = 997; **d**, *n* = 994; **e**, *n* = 947; **f**, *n* = 941; **g**, *n* = 987; **h**, *n* = 1,008; **i**, *n* = 994; **j**, *n* = 995; **k**, *n* = 992; **l**, *n* = 946; **m**, *n* = 940; **n**, *n* = 985. **m**, The association between tobacco use frequency and social anxiety did not survive Bonferroni correction. These results suggest a consistent association between more frequent substance use and greater psychiatric symptom burden in this population. Relationships between cannabis and tobacco frequency in the CHR group alone are reported in the Supplementary Information. Spearman correlations were calculated using two-sided tests across the full sample (CHR and healthy controls; *n* = 1,012). Shaded bands represent 95% confidence intervals around the linear regression line. Each dot represents an independent participant.

Symptom severity was assessed across seven domains: Scale of Psychosis-Risk Symptoms (SOPS) Positive, Negative, General and Disorganization; Calgary Depression Scale for Schizophrenia (CDSS), Self-Rating Anxiety Scale (SAS) and Social Interaction Anxiety Scale (SIAS). In the combined study population (that is, CHR and healthy controls), more frequent cannabis use was associated with greater psychiatric symptom severity on all domains (Bonferroni-corrected *P* = 0.05/seven symptom domains = 0.007, *P* < 0.001; Fig. 1). More frequent tobacco use was similarly associated with greater symptom severity across six of seven domains (all *P* < 0.001); however, the association with social anxiety did not survive Bonferroni correction (*ρ* = 0.08, *P* = 0.015; Fig. 1). See the Supplementary Information for details and analyses for the CHR group alone (Supplementary Fig. 6). In linear regression models, diagnosis and sex predicted psychiatric symptom severity, and diagnosis and tobacco or cannabis use predicted use of the other substance (Supplementary Results).

### Psychosis, depression and anxiety symptoms do not differ among CHR substance use groups

**Fig. 2:**
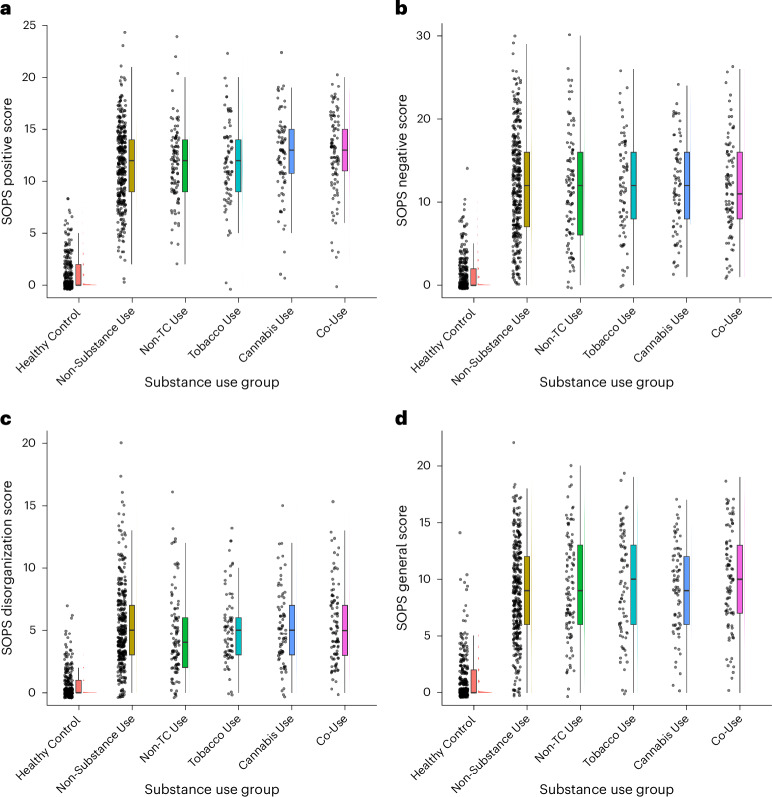
Psychosis symptoms do not differ among CHR substance use groups. **a**–**d**, SOPS positive (**a**), negative (**b**), disorganization (**c**) and general (**d**) scores across different substance use groups: Cannabis Use, Co-Use, Non-Substance Use, Non-TC Use, Tobacco Use and Healthy Controls. Group differences were assessed using one-way ANOVA with Bonferroni-corrected pairwise *t*-tests (two-sided): SOPS positive *F*(5,1,005) = 417.7, *P* < 0.001; SOPS negative *F*(5,991) = 156.2, *P* < 0.001; SOPS disorganization *F*(5,992) = 107.5, *P* < 0.001; SOPS general *F*(5,989) = 174.63, *P* < 0.001. Healthy Controls had significantly lower SOPS scores than all other groups (Non-Substance Use, Non-TC Use, Tobacco Use, Cannabis Use and Co-Use, Bonferroni-corrected *P* = 0.05/seven symptom domains = 0.007, *P* < 0.001), but their significance bars have been omitted for simplicity. No significant differences were observed between CHR substance use groups (all *P* > 0.05). Sample sizes varied slightly across panels due to missing data: **a**, *n* = 1,011; **b**, *n* = 997; **c**, *n* = 998; **d**, *n* = 995. Boxplots display the median (center line), 25th and 75th percentiles (box bounds), and 1.5× interquartile range (whiskers). Individual data points are shown as dots.

We then categorized CHR individuals based on their past 30-day substance use: (1) tobacco only; (2) cannabis only; (3) tobacco and cannabis co-use; (4) substance use, but neither tobacco nor cannabis (Non-TC use); or (5) no substance use). We observed that healthy controls had lower symptoms than all CHR substance use groups (all *P* < 0.001, Supplementary Information). There were no differences in symptoms (Fig. 2a–d, *P* > 0.05, Supplementary Fig. 8) among the CHR substance use groups.

### Survival analyses

We then tested whether the substance use pattern was associated with risk of conversion to psychosis. A total of 734 CHR participants provided data for survival analyses. Tobacco use frequency at baseline was not associated with risk of conversion to psychosis (hazard ratio (HR) = 1.12, 95% CI [0.90–1.40], *P* = 0.32; Supplementary Fig. 9). Details are provided in the Supplementary Information.

### Frequency of cannabis use alone is associated with higher risk of conversion

**Fig. 3:**
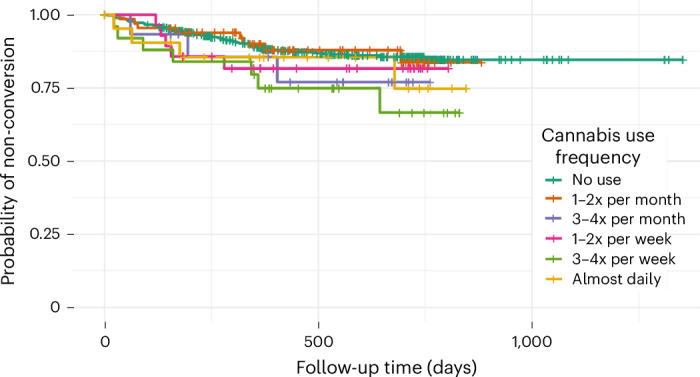
Cannabis use is associated with a higher risk of conversion to psychosis. The association between cannabis use frequency and conversion was assessed using a Cox proportional hazards model (two-sided), controlling for age and sex (*n* = 734 participants). Higher frequency of cannabis use at baseline was associated with higher risk of conversion to psychosis (HR = 1.17, 95% CI [1.02–1.34], *P* = 0.029). Neither age nor sex was associated with conversion (HR_age_ = 0.97, 95% CI [0.91–1.02], *P* = 0.216; HR_sex_ = 0.79, 95% CI [0.50–1.24], *P* = 0.298). Kaplan–Meier survival curves are shown for time to conversion to psychosis by ordinal cannabis use frequency. The curves are plotted for the purpose of descriptive survival patterns and are not adjusted for age or sex.

In Cox proportional hazard models adjusted for age and sex, a higher frequency of cannabis use at baseline was associated with higher risk of conversion to psychosis (HR = 1.17, 95% CI [1.02–1.34], *P* = 0.029; Fig. 3). The E-value for the point estimate was 1.61, and for the lower confidence interval limit was 1.14, indicating modest robustness to potential unmeasured confounding. Neither age nor sex was associated with conversion (HR_age_ = 0.97, 95% CI [0.91–1.02], *P* = 0.216; HR_sex_ = 0.79, 95% CI [0.50–1.24], *P* = 0.298).

### Cannabis use frequency is not associated with higher risk of conversion when controlling for tobacco use

To isolate the contributions of cannabis and tobacco use individually on conversion to psychosis, we performed analyses using cannabis or tobacco use frequency as a predictor while controlling for use of the other substance. Higher frequency of cannabis use remained positively associated with higher risk of conversion to psychosis (HR = 1.16, 95% CI [1.00–1.35], *P* = 0.055) when controlling for tobacco use, but the association was reduced and did not reach statistical significance. Neither age nor sex was associated with conversion (HR_age_ = 0.96, 95% CI [0.91–1.02], *P* = 0.211; HR_sex_ = 0.79, 95% CI [0.50–1.24], *P* = 0.305).

### Categorical tobacco and cannabis co-use is not significantly associated with higher risk of conversion to psychosis

When we defined tobacco and cannabis use categorically (no use, tobacco use only, cannabis use only, co-use), co-use of cannabis and tobacco was associated with higher risk of conversion compared to no use of either substance (HR = 1.69, 95% CI [0.96–2.97], *P* = 0.070, Supplementary Fig. 10), but did not reach significance. Details are provided in the Supplementary Information.

### Heavy cannabis use and light tobacco use is associated with the highest risk of conversion to psychosis

**Fig. 4:**
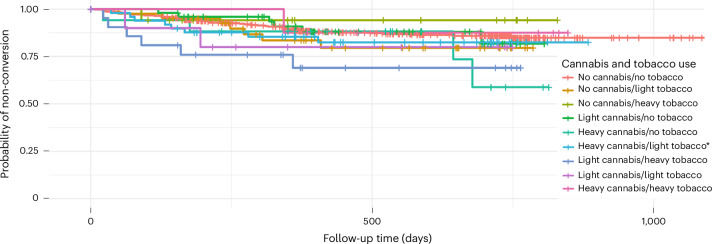
Heavy cannabis use and light tobacco use is associated with the highest risk of conversion to psychosis. To investigate the dose–response relationships of tobacco and cannabis use on conversion to psychosis, we categorized the intensity of cannabis and tobacco use (no use, light use, heavy use for each substance). The association between cannabis and tobacco use intensity and conversion to psychosis was assessed using a Cox proportional hazards model (two-sided), controlling for age and sex (*n* = 734 CHR participants). Heavy cannabis use and light tobacco use (HR = 2.93, 95% CI [1.23–6.97], *P* = 0.015) was associated with higher risk of conversion than no use of either substance. Neither age nor sex was associated with conversion (HR_age_ = 0.96, 95% CI [0.91–1.02], *P* = 0.216; HR_sex_ = 0.80, 95% CI [0.51–1.26], *P* = 0.330). Kaplan–Meier survival curves are shown for time to conversion to psychosis by categorical cannabis x tobacco interaction groups (none/low/high combinations). The curves are plotted for the purpose of descriptive survival patterns and are not adjusted for age or sex. The x axis is shortened for display purposes. *P < 0.05.

To investigate the dose–response relationships of tobacco and cannabis use on conversion to psychosis, we categorized the intensity of cannabis and tobacco use (no use, light use, heavy use for each substance; Supplementary Methods). Heavy cannabis use and light tobacco use (HR = 2.93, 95% CI [1.23–6.97], *P* = 0.015; Fig. 4) was associated with higher risk of conversion than no use of either substance. The corresponding E-value was 5.31, suggesting very strong robustness against unmeasured confounders. Neither age nor sex was associated with conversion (HR_age_ = 0.96, 95% CI [0.91–1.02], *P* = 0.216; HR_sex_ = 0.80, 95% CI [0.51–1.26], *P* = 0.330).

## Discussion

This Article describes an investigation of tobacco and cannabis co-use in individuals at CHR for psychosis. We observed associations between baseline tobacco and cannabis co-use and psychiatric symptom severity and increased risk of conversion to psychosis. We first investigated the frequency of substance use and observed that more frequent tobacco and cannabis use was associated with greater severity of psychosis, anxiety and depression. We then categorized CHR individuals based on whether they used tobacco or cannabis in isolation or together (co-use), and found psychiatric symptom severity did not differ between substance use groups (that is, CHR individuals with co-use did not have more severe symptoms than those with isolated tobacco or cannabis use). These results were contrary to our hypotheses and suggest that tobacco and cannabis use do not have additive effects on clinical symptomatology. We also did not observe any beneficial effects of tobacco or cannabis use on symptoms. Next, we investigated the effects of co-use on conversion to psychosis and observed that heavy co-use (heavy cannabis and light tobacco use) was associated with a threefold increased risk of conversion to psychosis.

Our findings dovetail with mixed reports in the literature. Nicotine transiently improves cognitive deficits and negative symptoms in schizophrenia, probably via agonism of nicotinic acetylcholine receptors to increase dopamine and glutamate transmission in the brain^8,37,38^. However, heavy nicotine use has been associated with worse outcomes in chronic psychosis, as individuals who smoke more heavily have more severe positive and negative symptoms, more frequent hospitalizations, and higher relapse rates^8^. Cannabis use exhibits a similarly paradoxical profile: acutely, some individuals report anxiolytic or mood-elevating effects from cannabis, yet sustained use is associated with worsened symptom severity, poorer treatment adherence and poorer functional outcomes^39^. Yet, previous research has observed that CHR individuals who used cannabis had lower social anhedonia and higher social engagement compared to those who did not use cannabis^40^. Our observation that symptom severity did not differ among the CHR substance use groups contrasts with a recent study of FEP individuals that found individuals who used only tobacco prior to psychosis onset had less severe negative symptoms compared to those who did not use tobacco^34^. Additionally, FEP individuals who used tobacco and cannabis before psychosis onset had more severe positive symptoms than individuals who only used tobacco^34^. However, this analysis measured symptoms during FEP rather than the CHR period and assessed substance use retrospectively^34^.

Another critical aspect is why individuals at risk for psychosis are at greater risk for substance use. Those who develop schizophrenia are four to five times more likely to have had substance use disorders in their youth than the general population^40^. In CHR cohorts, problematic substance use occurs in 22% to >50% of individuals and is significantly higher than in age-matched peers. One proposed mechanism is the self-medication hypothesis: adolescents in the prodromal phase often experience subthreshold symptoms of psychosis and may use substances to cope with distressing experiences^39^. Another important factor is shared neurobiological and genetic vulnerability. Individuals with heightened dopaminergic reactivity might be more prone to psychotic experiences and also more sensitive to the reinforcing effects of substances, making them more likely to use substances^40,41^. Thus, the high co-occurrence of psychosis risk status and substance use probably arises from a confluence of factors: attempts to quell emerging symptoms, overlapping biological susceptibilities, and lifestyle or social factors that provide access to substances.

Critically, in a model differentiating light versus heavy use, individuals with heavy cannabis and light tobacco use had the highest conversion risk—a threefold increase. Our findings indicate that co-use of tobacco and cannabis is a more potent risk factor for psychosis conversion than use of either substance alone. However, the directionality of this relationship cannot be known. One possible interpretation is a synergistic effect, where tobacco might enhance the impact of cannabis use on the brain’s dopaminergic or endocannabinoid systems, thereby amplifying the biological processes underlying psychosis onset. Acutely, both substances increase dopamine signaling in the mesolimbic pathway by increasing the firing of ventral tegmental area dopamine neurons and elevating dopamine release in the nucleus accumbens^42,43^. Cannabis and tobacco may sensitize dopamine neurotransmission and can alter mesocorticolimbic dopamine signaling with chronic use^18,44^. Nicotine use may predispose to cannabis use or potentiate its effects by increasing tetrahydrocannabinol (THC) absorption^45^. A recent study showed that cannabis and tobacco co-use was associated with increased levels of fatty-acid amide hydrolase, which degrades anandamide, a prominent endocannabinoid^46^. Additionally, smoking nicotine and cannabis simultaneously increases the amount of THC inhaled per gram^47^. This theory also aligns well with evidence from FEP cohorts. In a large multinational study of FEP, co-use of tobacco and cannabis predicted earlier age of FEP onset than either alone, but co-use did not differ from tobacco-only use in the odds of developing psychosis^33^. Rather than being directly causal, evidence suggests tobacco and cannabis co-use may be a marker of elevated underlying vulnerability. Heavy cannabis use combined with light tobacco use conferred the highest risk of conversion. An elevated risk at relatively low levels of tobacco exposure may argue against a dose-dependent tobacco effect and instead suggests that tobacco use may act as a sensitizing factor or marker of heightened underlying susceptibility in the context of frequent cannabis use.

However, cannabis use appears to be the primary culprit, as cannabis use predicted conversion to psychosis, although this relationship was attenuated after controlling for tobacco use. These results extend prior research on cannabis use in CHR populations. Cannabis use is linked to increased psychosis risk across multiple epidemiological studies. Previous longitudinal research has shown that heavy cannabis use in adolescence substantially raises the likelihood of later developing schizophrenia-spectrum disorders^48^. Individuals reporting very frequent cannabis use (50+ times by late teens) have up to a sixfold higher odds of a schizophrenia diagnosis in adulthood compared to individuals without substance use^48^. Cannabis use disorder is also linked to increased dopamine-related function in a midbrain pathway implicated in psychosis^41^. Likewise, meta-analyses have concluded that, although cannabis is neither a necessary nor sufficient cause of psychosis, it probably contributes to psychosis risk in predisposed individuals^48^.

### Strengths and limitations

Our study has several notable strengths. This is the first investigation examining tobacco and cannabis co-use in a CHR cohort. Earlier work in psychosis risk largely focused on single substances, whereas our approach captures the real-world scenario where many young people use tobacco and cannabis together. Although many cross-sectional studies assess current or lifetime substance use disorders, few studies characterize current substance use below the level of a disorder. Our sample is large (734 CHR individuals, from an initial pool of 1,012 participants including controls) and drawn from eight sites across North America, enhancing generalizability.

Although our study sheds new light on tobacco and cannabis co-use in the CHR period, it has several limitations. A major limitation is that the data were collected from 2009 to 2013, before the legalization of recreational cannabis use, the emergence of vaping (nicotine and THC)^49,50^ and other non-combustible nicotine use, and increasing levels of THC, which is important as THC potency has increased since 2013^51,52^. The available substance use data were limited in detail, relying on self-reported current substance use without data on quantity or use history. Furthermore, the substance use data lacked granularity to differentiate the effects of simultaneous versus asynchronous use of tobacco and cannabis, which may have differing neurobiological effects. Individuals with a substance use disorder in the past six months were excluded, which also limits the generalizability of our findings. Finally, the observational design precludes conclusions about causality, and we did not include longitudinal assessments in this analysis. Although co-use predicted conversion to psychosis, we cannot be certain that substance use is driving this outcome. It is possible that a shared third factor (for example, genetic vulnerability or childhood trauma) leads some CHR individuals to both use substances and develop psychosis.

### Future directions

Given the scarcity of literature on tobacco and cannabis co-use in psychosis risk, our findings expand several avenues for future research. Replication in more recent, independent samples will be important to confirm the heightened conversion risk among individuals who co-use tobacco and cannabis. From a clinical perspective, our findings suggest that interventions to reduce substance use in CHR populations are warranted. If co-use indeed contributes to conversion risk, then reducing substance use might delay or prevent psychosis onset in some individuals. In conclusion, our study underscores that tobacco and cannabis co-use is an important, yet understudied, factor in the psychosis prodrome.

## Materials and methods

### Participants

NAPLS2 is a longitudinal case–control study of individuals at CHR for psychosis across eight sites in North America. Individuals meeting CHR criteria (*N* = 734) and healthy controls (*N* = 278) were enrolled. The Supplementary Information describes the inclusion and exclusion criteria. Participants underwent assessment at baseline and every six months for two years and upon conversion to psychosis (if applicable) from January 2009 to April 2013. Participant demographics, including race and ethnicity, were self-reported. Only baseline assessments were used for this analysis. Before participation, all participants provided written informed consent (or, if under 18 years of age, informed assent with parental consent) in accordance with the institutional review boards of Beth Israel Deaconess Medical Center (Boston, Massachusetts), Emory University (Atlanta, Georgia), the University of Calgary (Alberta, Canada), University of California Los Angeles, University of California San Diego, The University of North Carolina at Chapel Hill, Yale University (New Haven, Connecticut) and Zucker Hillside Hospital (New York) (see Supplementary Information for details).

### Measures

#### Diagnosis

CHR individuals met the Criteria for the Psychosis Risk Syndrome^53^ based on the Structured Interview for Psychosis-Risk Syndrome (SIPS)^53^. Conversion to psychosis was determined in a follow-up SIPS interview, defined as meeting the Presence of Psychosis Syndrome criteria.

#### Substance use

Current substance use at baseline was measured using the Alcohol Use Scale/Drug Use Scale (AUS/DUS), which assesses substance use frequency over the past 30 days on an ordinal scale^54^ (Supplementary Information). We also categorized CHR individuals into groups based on their reported substance use in the past 30 days: (1) tobacco only; (2) cannabis only; (3) tobacco and cannabis co-use; (4) substance use, but neither tobacco nor cannabis (Non-TC use); (5) no substance use. Healthy controls had minimal substance use and so were treated as one group. Because these co-use categories did not consider intensity of use, tobacco and cannabis use were also treated as individual ordinal variables to test for dose–response relationships.

#### Psychiatric symptoms

The severity of psychosis symptoms at baseline was rated using the SOPS^53^. Anxiety and depressive symptoms were measured with the SAS^55^, SIAS^56^ and CDSS^57^.

### Statistical approach

We used *t*-tests to compare continuous outcomes based on dichotomous variables. ANOVAs were used to compare continuous outcomes (for example, symptom scores) based on three or more groups. Kruskal–Wallis tests were used to compare differences in ordinal variables (for example, substance use frequency) across substance use groups. Spearman correlations were used to determine relationships with ordinal variables. Linear regression models were used to predict (1) symptom severity and (2) substance use frequency based on age, sex, site, diagnosis and diagnosis x substance use interactions. All analyses were conducted in RStudio (version 2023.03.1 + 446) using alpha < 0.05, except as indicated in the results, to correct for multiple comparisons.

#### Survival analyses

Cox proportional hazards regression models were used to examine the association between tobacco and cannabis use and conversion to psychosis. Time was measured as days from initial assessment to last follow-up assessment or post-conversion assessment. We fitted five Cox proportional hazards models controlling for age and sex:

1. Tobacco use (ordinal)
2. Cannabis use (ordinal)
3. Cannabis and tobacco simultaneously (both ordinal)
4. Categorical co-use (no use, tobacco use only, cannabis use only, co-use)
5. Categorical intensity of co-use (no use, light use, heavy use for tobacco and cannabis).

The reference category for the categorical models was no use of either substance. Consistent with earlier literature^26,30^, we defined light tobacco use as <10 per day; heavy tobacco use as >10 per day, light cannabis use as up to twice per week, and heavy cannabis use as three or more times per week (Supplementary Information). Hazard ratios with 95% confidence intervals were calculated for each predictor. All analyses were conducted using R version 4.5.1 (2025-06-13) and the packages survival and survminer. We calculated E-values for the substance use variables in the Cox models to quantify the minimum strength of unmeasured confounding needed to explain the observed associations.

### Reporting Summary

Further information on research design is available in the Nature Portfolio Reporting Summary linked to this Article.

## Supplementary information

Supplementary Information Supplementary Methods, Results, Table 1 and Figs. 1–10.

Reporting Summary

## Data Availability

Data is available upon reasonable request.
