# Supplementary material for: Cannabis and tobacco co-use predicts psychosis in clinical high risk cohorts
Source: Nat Ment Health. 2026 May 12;4(6):941–50. doi: 10.1038/s44220-026-00648-y (PMC13259916; doi:10.1038/s44220-026-00648-y)
Supplement: Supplementary file 1 — Supplementary Methods, Results, Table 1 and Figs. 1–10. [file 44220_2026_648_MOESM1_ESM.pdf]

nature mental health

Supplementary information https://doi.org/10.1038/s44220-026-00648-y

Cannabis and tobacco co-use predicts psychosis in clinical high risk cohorts

In the format provided by the authors and unedited

Supplemental Material

Supplemental Methods

Participants

Inclusion and Exclusion Criteria

Participants underwent clinical assessment at baseline and every 6 months for two years and upon conversion to psychosis (if applicable) from January 2009 to April 2013. Prior to participation, all participants provided written informed consent (or, if under age 18, informed assent with parental consent) in accordance with the institutional review boards of Beth Israel Deaconess Medical Center, Boston, Massachusetts; Emory University, Atlanta, Georgia; University of Calgary, Alberta, Canada; University of California, Los Angeles; University of California, San Diego; The University of North Carolina at Chapel Hill; Yale University, New Haven, Connecticut; and Zucker Hillside Hospital, New York.

CHR individuals were included if they met the Criteria for the Psychosis Risk Syndrome,[1] based on the Structured Interview for Prodromal Symptoms (SIPS).[2] If individuals were younger than 19 years, they were included based on criteria for schizotypal personality disorder or Criteria for the Psychosis Risk Syndrome. Individuals could meet any of four prodromal criteria: 1) attenuated positive symptoms (APS), 2) brief intermittent psychotic symptoms (BIPS), 3) genetic risk and deterioration (GRD), or 4) youth and schizotypy criteria (YS). A CHR participant could meet criteria for multiple categories. The Structured Clinical Interview for DSM was used to exclude psychosis and to identify DSM-IV Axis I or cluster A personality disorders. Anyone with a lifetime Axis I psychotic disorder, estimated IQ less than 70 on both measures of IQ, a central nervous system disorder, or DSM-IV substance dependence in the past 6 months was excluded. Other nonpsychotic DSM-IV disorders were not exclusionary (e.g. depression, substance use disorders) unless they clearly caused or better explained prodromal symptoms. CHR individuals were permitted to take antipsychotic medication as long as they had not developed any psychotic symptoms prior to initiating medication. Healthy controls were not permitted to meet any prodromal criteria, have a history of a psychotic or cluster A personality disorder, or have a family history of a psychotic disorder in a first-degree relative.

Measures

Substance Use: Current substance use was measured using the Alcohol Use Scale/Drug Use Scale (AUS/DUS), which assesses substance use severity (abstinent, use without impairment, abuse, dependence, dependence with institutionalization) and frequency over the past 30 days for the following substances: tobacco, alcohol, marijuana, cocaine, opiates, phencyclidine (PCP), amphetamines, methylenedioxymethamphetamine (MDMA), gamma-hydroxybutarate, huffing, hallucinogens, and other substances.[3] Substance use frequency was measured on an ordinal scale for tobacco in cigarettes per day (0 = no use, 1 = occasionally, 2 = less than 10 per day, 3 = 11-25 per day, 4 = more than 25 per day) and for all other substances (0 = no use, 1 = once or twice per month, 2 = 3-4 times per month, 3 = 1-2 times per week, 4 = 3-4 times per week, 5 = almost daily). CHR individuals were categorized into one of 5 groups based on their reported substance use in the past 30 days: 1) Tobacco only; 2) Cannabis only; 3) Tobacco and Cannabis Co-Use; 4) Neither Tobacco nor Cannabis (Non-TC users); and 5) No substance use. Healthy controls had minimal substance use and so were treated as one group. A total of 734 CHR individuals had substance use data and were included in the analysis alongside 278 healthy controls.

Survival Analyses

Cox proportional hazards regression models were used to examine the association between tobacco and cannabis use and conversion to psychosis. Time was measured as days from initial assessment to last follow up assessment or post-conversion assessment. We fitted five Cox proportional hazards models controlling for age and sex:

1. Tobacco use (ordinal)
2. Cannabis use (ordinal)
3. Cannabis and Tobacco simultaneously (both ordinal)
4. Categorical Co-Use (No use, Tobacco use only, Cannabis use only, Co-Use)
5. Categorical Intensity of Co-Use (No use, Light use, Heavy use for Tobacco and Cannabis)

For the Categorical Co-Use survival analyses, CHR individuals were categorized into one of 4 groups based on their reported substance use in the past 30 days: 1) Neither Tobacco nor Cannabis use; 2) Tobacco use only; 3) Cannabis use only; and 4) Tobacco and Cannabis Co-Use.

For the Categorical Intensity of Co-Use, CHR individuals were categorized based on their patterns of tobacco and cannabis use in the past 30 days: 1) No use; 2) Light use; or 3) Heavy use. Consistent with prior literature,[4,5] we defined light tobacco use as <10 per day (1 or 2 on AUS/DUS); heavy tobacco use as >10 per day (3 or 4 on AUS/DUS), light cannabis use as up to twice per week (1, 2, or 3 on AUS/DUS), and heavy cannabis use as three or more times per week (4 or 5 on AUS/DUS).

Psychiatric Symptoms: Severity of positive, negative, disorganized and general symptoms was rated on the Scale of Psychosis-Risk Symptoms (SOPS).[1] The SOPS is a clinician-administered assessment tool designed to evaluate early signs of psychosis in individuals at clinical high risk (CHR). Developed as part of the Structured Interview for Prodromal Syndromes (SIPS), the SOPS consists of four symptom domains: positive symptoms (e.g., unusual thought content, suspiciousness, perceptual abnormalities), negative symptoms (e.g., social withdrawal, decreased motivation), disorganized symptoms (e.g., trouble with communication and thinking), and general symptoms (e.g., anxiety, sleep disturbances). Anxiety and depressive symptoms were measured through the Self-Rating Anxiety Scale (SAS),[6] Social Interaction Anxiety scale (SIAS),[7] and the Calgary Depression Scale for Schizophrenia (CDSS).[8] The SAS is a self-report questionnaire designed to assess the severity of anxiety symptoms in individuals.[6] The SIAS is a self-report questionnaire that assesses anxiety experienced in social interactions.[7] The CDSS is a clinician-administered tool designed to assess depressive symptoms specifically in individuals with schizophrenia.[8]

Race and Ethnicity: Race and ethnicity were self-reported.

Supplemental Results

Demographic Differences in Tobacco and Cannabis Use

Across all subjects, men had a higher frequency of cannabis use ($H(1)=8.412$, $p=.0037$) and tobacco use ($H(1)=10.17$, $p=.0014$) than women (Supplemental Figures 4-5).

Greater Tobacco and Cannabis Use are Associated with Greater Psychiatric Symptom Severity

In the combined study population (i.e., CHR and healthy controls), more frequent cannabis and tobacco use was associated with greater psychiatric symptom severity (Bonferroni-corrected p=.05/7 symptom domains=0.007). More frequent cannabis use was associated with greater severity across all four SOPS symptom domains, including positive (Spearman $\rho = 0.20$, $p<.001$, Figure 1A), negative (Spearman $\rho = 0.11$, $p<.001$, Figure 1B), disorganization (Spearman $\rho = 0.14$, $p<.001$, Figure 1C), and general symptoms (Spearman $\rho = 0.17$, $p<.001$, Figure 1D), as well as elevated anxiety (Spearman $\rho = 0.15$, $p<.001$, Figure 1E), social anxiety (Spearman $\rho = 0.11$, $p=.0011$, Figure 1F), and depression (Spearman $\rho = 0.15$, $p<.001$, Figure 1G).

Similarly, higher tobacco use frequency was associated with higher symptom severity across 6 of 7 clinical measures (Bonferroni-corrected p=.05/7 symptom domains=0.007), including positive (Spearman $\rho = 0.17$, $p<.001$, Figure 1H), negative (Spearman $\rho = 0.13$, $p<.001$, Figure 1I), disorganization (Spearman $\rho = 0.16$, $p<.001$, Figure 1J), and general psychosis symptoms (Spearman $\rho = 0.19$, $p<.001$, Figure 1K), anxiety (Spearman $\rho = 0.19$, $p<.001$, Figure 1L), and depression severity (Spearman $\rho = 0.16$, $p<.001$, Figure 1N). The association between tobacco use frequency and social anxiety did not survive Bonferroni correction (Spearman $\rho = 0.08$, $p=.015$, Figure 1M).

In Individuals at CHR, Greater Tobacco and Cannabis Use are Associated with Greater Psychiatric Symptom Severity

In the CHR population alone, more frequent cannabis and tobacco use was associated with greater psychiatric symptom severity (Bonferroni-corrected p=.05/7 symptom domains=0.007). More frequent cannabis use was associated with greater positive symptoms (Spearman $\rho = 0.13$, $p=.0004$, Supplemental Figure 6A). However, more frequent cannabis use was not associated with negative (Spearman $\rho = -0.015$, $p=.69$, Supplemental Figure 6B), disorganization (Spearman $\rho = 0.046$, $p=.22$, Supplemental Figure 6C), or general psychosis symptoms (Spearman $\rho = 0.071$, $p=.056$, Supplemental Figure 6D), or anxiety (Spearman $\rho = 0.038$, $p=.32$, Supplemental Figure 6E), social anxiety (Spearman $\rho = -0.005$, $p=.89$, Supplemental Figure 6F), or depression (Spearman $\rho = 0.061$, $p=.10$, Supplemental Figure 6G).

Higher tobacco use frequency was associated with higher anxiety severity (Spearman ρ = 0.106, p=.0054, Supplemental Figure 6L, Bonferroni-corrected p=.05/7 symptom domains=0.007). Tobacco use frequency was not associated with positive (Spearman ρ = 0.06, p=.10, Supplemental Figure 6H), negative (Spearman ρ = 0, p=.99, Supplemental Figure 6I), disorganization (Spearman ρ = 0.047, p=.20, Supplemental Figure 6J), or general psychosis symptoms (Spearman ρ = 0.093, p=.014, Supplemental Figure 6K), or social anxiety (Spearman ρ = -0.043, p=.26, Supplemental Figure 6M), or depression severity (Spearman ρ = .061, p=.10, Supplemental Figure 6N).

To examine relationships with cannabis use frequency in the CHR sample, we ran a linear regression model predicting cannabis use frequency based on age, sex, site, and tobacco use frequency without the effect of diagnosis (F(10,723)=11.52, p<.001). In this model of only individuals at CHR, male sex (Estimate= -0.228, SE=0.091, t=-2.514, p=.012), more frequent tobacco use (Estimate=0.412, SE=0.050, t=8.182, p<.001), and the Georgia site (Estimate=0.5792, SE=0.1886, t=3.071, p=.0022) predicted more frequent cannabis use.

To examine relationships with tobacco use frequency within the CHR sample, we ran a linear regression model predicting tobacco frequency based on age, sex, site, and cannabis use frequency without the effect of diagnosis (F(10,723)=12.31, p<.001). In this model of only individuals at CHR, older age (Estimate=0.0284, SE=0.007939, t=3.582, p<.001), male sex (Estimate= -0.132, SE=0.06425, t=-2.049, p=.041), more frequent cannabis use (Estimate=0.206, SE=0.0251, t=8.182, p<.001), the Georgia (Estimate=0.2843, SE=0.1337, t=2.126, p=.034), New York (Estimate=0.2918, SE=0.1252, t=2.332, p=.020), North Carolina (Estimate=0.3266, SE=0.1252, t=2.608, p=.0093), and Calgary sites (Estimate=0.2935, SE=0.109, t=2.686, p=.0074) were significant predictors of more frequent tobacco use.

Diagnosis and Sex Predict Psychosis Symptom Severity

We performed linear regression models predicting psychosis symptom severity based on age, sex, study site, cannabis use frequency, tobacco use frequency, diagnosis (CHR or healthy control), the diagnosis*cannabis frequency interaction, the diagnosis*tobacco frequency interaction, and the cannabis frequency*tobacco frequency interaction.

In a model predicting SOPS positive symptoms (F(15,992)=150.3, p<.001), CHR diagnosis (Estimate=10.84, SE=0.260, t=41.768 p<0.001) and the Georgia site (Estimate=1.6586, SE=0.43105, t=3.848, p<.001) predicted higher scores, while the San Diego site predicted lower scores (Estimate= -1.32557, SE=0.39941, t=-3.319, p<.001).

In a model predicting SOPS negative symptoms (F(15,978)=57.12, p<.001), CHR diagnosis (Estimate=10.74, SE=,0.415, t=25.8782, p<.001), while male sex (Estimate=-1.1056, SE=0.337, t=-3.276, p<.001), and the Boston (Estimate=1.771417, SE=0.7086, t=2.5, p=.013) and New York sites (Estimate=2.004661, SE=0.651242, t=3.078, p=.0021) predicted higher scores.

In a model predicting SOPS general symptoms (F(15,976)=72.31, p<.001), CHR diagnosis (Estimate=7.99, SE=0.286, t=27.936, p<.001), female sex (Estimate=1.077, SE=0.233, t=4.626, p<.001), the New York (Estimate=2.42586, SE=0.44979, t=5.393, p<.001) and Connecticut sites (Estimate=2.83278, SE=0.44079, t=6.427, p<.001), and higher cannabis use frequency (Estimate=0.856, SE=0.291, t=2.942, p=.0033) predicted higher scores, while the interaction between higher cannabis use frequency and CHR diagnosis predicted lower general symptom scores (Estimate= -0.685, SE=0.322, t=-2.129, p=.033).

In a model predicting SOPS disorganization symptoms (F(15,979)=37.83, p<.001), CHR diagnosis predicted higher scores (Estimate = 4.521, SE = 0.219, t=20.643, p<.001), while the San Diego (Estimate= -0.887335, SE=0.341993, t=-2.595, p=.0096) and Calgary sites (Estimate= -0.637002, SE=0.316625, t=-2.012, p=.045) predicted lower disorganization scores.

Diagnosis, Age, Sex, Tobacco and Cannabis Use Frequency Predict Anxiety and Depression Symptoms

Linear regression models were also used to predict anxiety (SAS), social anxiety (SIAS), and depression (CDSS) based on age, sex, study site, cannabis use frequency, tobacco use frequency, diagnosis (CHR or healthy control), the diagnosis*cannabis frequency interaction, the diagnosis*tobacco frequency interaction, and the cannabis frequency*tobacco frequency interaction.

In a model predicting anxiety severity (F(15,930)=42.35, p<0.001, CHR diagnosis (Estimate=14.632, SE=0.739, t=19.794, p<.001), female sex (Estimate=4.434, SE=0.596, t=7.445, p<.001), the Calgary site (Estimate=4.510211, SE=1.087989, t=4.145, p<.001), and higher cannabis use frequency (Estimate=1.722, SE=0.730, t=2.359, p=.019) predicted greater anxiety.

In a model predicting social anxiety severity, (F(15,924)=27.06, p<0.001, CHR diagnosis (Estimate=23.290, SE=1.272, t=18.311, p<.001) and older age (Estimate=0.473, SE=0.123, t=3.862, p<.001) predicted higher scores.

In a model predicting depression severity, (F(15,969)=27.87, p<0.001, CHR diagnosis (Estimate=5.438; SE=0.321; t=19.951; p<.001), female sex (Estimate=0.654, SE=0.260, t=2.513, p=.012), older age (Estimate=0.163, SE=0.03127, t=5.214, p<.001), and the New York site (Estimate=1.89586, SE=0.50541, t=3.751, p<.001) predicted higher scores, while the North Carolina site (Estimate= -1.06914, SE=0.51524, t=-2.075, p=.038) predicted lower depression scores.

Diagnosis and Tobacco Use Predict Cannabis Use

Linear regression models were used to predict cannabis use frequency based on age, sex, site, diagnosis, tobacco frequency, and diagnosis*tobacco use frequency (F(12,996)=13.23, p<.001). Male sex (Estimate=-0.189, SE=0.070, t=-2.695, p=.0072), CHR diagnosis (Estimate=0.191, SE=0.0836, t=2.287, p=.022), and the Georgia site (Estimate=0.397, SE=0.1434, t=2.768, p=.0057) were significant predictors of cannabis use. The diagnosis*tobacco use interaction (Estimate=0.371. SE=0.146, t=2.537, p=.011) predicted more frequent cannabis use such that CHR participants who used tobacco more frequently also used cannabis more frequently (Supplemental Figure 7).

Diagnosis and Cannabis Use Predict Tobacco Use

Linear regression models were used to predict tobacco use frequency based on age, sex, site, diagnosis, cannabis use frequency, and diagnosis*cannabis use frequency (F(12,996)=15.97, p<.001). Older age (Estimate=0.0262, SE=0.0058, t=4.51, p<.001), CHR diagnosis (Estimate=0.224, SE=0.058, t=3.866, p<.001), male sex (Estimate=-0.109, SE=0.0489, t=-2.225,

p=.026), and the diagnosis*cannabis use frequency interaction (Estimate=0.157, SE=0.0654, t=2.404, p=.016) were predictors of tobacco use such that CHR participants with more frequent cannabis use also had more frequent tobacco use (Supplemental Figure 7). The Georgia (Estimate=0.2324, SE=0.0999, t=2.325, p=.020), New York (Estimate=0.203, SE=0.0933, t=2.177, p=.030), North Carolina (Estimate=0.224073, SE=0.095, t=2.358, p=.0186), and Calgary sites (Estimate=0.2365, SE=0.0853, t=2.771, p=.0057) were all predictors of greater tobacco use frequency.

Tobacco and Cannabis Use are Associated with Prodromal Diagnostic Criteria

As an exploratory analysis, we tested if prodromal diagnostic criteria (APS, BIPS, GRD, YS) predicted cannabis use in the CHR group (F(4,730)=3.127, p=.014). Individuals who met GRD criteria had higher cannabis use frequency (Estimate=0.3847, SE=0.1660, t=2.318, p=.021). We then included the prodromal diagnostic criteria into a model alongside age, sex, site, and tobacco use frequency (F(14,719)=8.937, p<.001). In this model, meeting GRD criteria (Estimate=0.346, SE=0.158, t=2.187, p=.029), the Georgia site (Estimate=0.6536, SE=0.1905, t=3.431, p=.00064), and higher tobacco frequency (Estimate=0.4013, SE=0.0505, t=7.939, p<.001) were significant predictors of cannabis use.

We then tested if prodromal diagnostic criteria predicted tobacco use frequency in the CHR sample (F(4,730)=3.09, p=.015). Individuals who met APS (Estimate=-0.3511, SE=0.145, t=-2.415, p=.016) criteria used tobacco less frequently. When we included prodromal diagnostic criteria alongside age, sex, site, and cannabis use frequency (F(14,719)=9.331, p<.001), older age (Estimate=0.0282, SE=0.008235, t=3.423, p<.001), individuals who met APS criteria (Estimate=-0.317, SE=0.137, t=-2.318, p=.021), and higher cannabis use frequency (Estimate=0.200834, SE=0.0253, t=7.939, p<.001) were significant predictors of tobacco use such that older age and more frequent cannabis use predicted more frequent tobacco use, while meeting APS criteria predicted less frequent tobacco use. In addition, the Georgia (Estimate=0.28805, SE=0.135418, t=2.127, p=.034), New York (Estimate=0.298937, SE=0.125253, t=2.387, p=.017), North Carolina (Estimate=0.339443, SE=0.125407, t=2.707, p=.0070), Calgary (Estimate=0.301906, SE=0.110031, t=2.744, p=.0062), and Connecticut sites (Estimate=0.245233, SE=0.122051, t=2.009, p=.045) were all predictors of greater tobacco use frequency.

Healthy Controls have Lower Symptoms than All CHR Substance Use Groups

We investigated if symptom severity differed by substance use group. Symptom severity was assessed across 7 domains: SOPS Positive, Negative, General, and Disorganization; CDSS, SAS, and SIAS. Healthy controls had lower psychosis symptom severity than all CHR substance use groups (Bonferroni-corrected p=.05/7 symptom domains=0.007, SOPS Positive F(5,1,005)=417.7, p<.001, Figure 2A; SOPS Negative F(5,991)=156.2, p<.001, Figure 2B; SOPS General F(5,989)=174.63, p<.001, Figure 2C; SOPS Disorganization F(5,992)=107.5, p<.001, Figure 2D). Healthy Controls had lower anxiety (SAS, F(5,942)=97.81, p<.001), social anxiety (SIAS, F(5,936)=75.44, p<.001), and depression (CDSS, F(5,982)=68.78, p<.001, Supplemental Figure 8) scores than all CHR substance use groups.

Psychiatric Symptoms Do Not Differ Among CHR Substance Use Groups

Among the CHR substance use groups (Tobacco-only, Cannabis-only, Co-Use, Non-TC, No substance use), there were no differences in psychosis symptoms (Bonferroni-corrected p=.05/7 symptom domains=0.007, SOPS Positive F(4)=2.10, p>.05; SOPS Negative F(4)=0.18, p>.05, SOPS General F(4)=1.66, p>.05, SOPS Disorganization F(4)=1.21, p>.05) or in anxiety (F(4)=2.11, p>.05), social anxiety (F(4)=0.90, p>.05), or depression (F(4)=2.88, p=.02).

Survival Analyses

Survival analyses were performed in a subset of 734 participants, including just the CHR individuals, with complete data. This subset had a mean age of 18.47 years (SD = 4.23). The majority (57.63%) were male, and 83 of these individuals converted to psychosis at follow up (11.31%). For individuals who had an unknown survival time, we replaced their follow up days number with 0, and they were categorized as non-convertors. In this sample, 480 individuals reported no tobacco or cannabis use, 78 individuals reported tobacco use only, 79 individuals reported cannabis use only, and 97 individuals reported using both tobacco and cannabis.

Frequency of Tobacco Use Alone is Not Associated with Higher Risk of Conversion

Tobacco use frequency at baseline was not significantly associated with higher risk of conversion to psychosis (*HR* = 1.12, 95% CI [0.90–1.40], *p* = .32, Supplemental Figure 9). Neither age nor sex were associated with conversion (*HRage* = 0.97, 95% CI [0.92‐1.03], *p* = 0.290; *HRsex* = 0.76, 95% CI [0.48‐1.19], *p* = 0.227).

Categorical Tobacco and Cannabis Co-Use is Not Significantly Associated with Higher Risk of Conversion to Psychosis

Although baseline symptom levels did not differ among CHR substance use groups, we next investigated whether cannabis and tobacco use (alone and co-use) was associated with increased risk of conversion to psychosis. When we defined tobacco and cannabis use categorically (no use, tobacco use only, cannabis use only, co-use), co-use of cannabis and tobacco was associated with higher risk of conversion compared to no use of either substance (*HR* = 1.69, 95% CI [0.96–2.97], *p* = .070, Supplemental Figure 10) but did not reach significance. Cannabis use only (*HR* = 1.29, 95% CI [0.64–2.57], *p* = .47) and tobacco use only (*HR* = 1.25, 95% CI [0.58–2.67], *p* = .57) did not significantly predict conversion compared to no use of either substance. Neither age nor sex were associated with conversion (*HRage* = 0.97, 95% CI [0.91‐1.02], *p* = 0.238; *HRsex* = 0.78, 95% CI [0.49‐1.22], *p* = 0.274).

Supplemental Tables & Figures

Supplemental Table 1. NAPLS2 Demographics

	**CHR**			**Healthy Control (n=278)**
	**CHR (n=734)**	**CHR-NC (n=651)**	**CHR-C (n=83)**	
Age, y (SD)	18.6 (4.3)*	18.5 (4.3)	18.1 (3.6)	19.7 (4.7)
Sex, Male (%)	423 (57.6)	370 (56.8)	53 (63.9)	140 (50.4)
Race				
First Nations (%)	13 (1.7)	12 (1.8)	1 (1.2)	4 (1.4)
East Asian (%)	19 (2.5)	18 (2.8)	1 (1.2)	15 (5.4)
Southeast Asian (%)	15 (2.0)	12 (1.8)	3 (3.6)	7 (2.5)
South Asian (%)	19 (2.6)	16 (2.5)	3 (3.6)	8 (2.9)
Black (%)	109 (14.9)	99 (15.2)	10 (12.1)	48 (17.3)
Central/South American (%)	32 (4.4)	28 (4.3)	4 (4.8)	13 (4.6)
West/Central Asia and Middle East (%)	6 (0.8)	5 (0.8)	1 (1.2)	2 (0.7)
White (%)	426 (58.0)	379 (58.2)	47 (56.6)	151 (54.3)
Native Hawaiian or Pacific Islander (%)	2 (0.3)	1 (0.2)	1 (1.2)	1 (0.4)
Interracial (%)	92 (12.5)	80 (12.3)	12 (14.5)	29 (10.4)
Hispanic (%)	138 (18.8)	123 (18.9)	15 (18.1)	49 (17.6)
Taking Antipsychotic (%)	58 (7.9)	50 (7.7)	8 (9.6)	-

CHR: clinical high risk; CHR-NC: clinical high risk – nonconverter; CHR-C: clinical high risk – converter; HC: healthy control. Comparisons were made between 1) CHR and healthy controls and 2) between CHR-C and CHR-NC. There were no significant differences in demographic variables between CHR-C and CHR-NC. ***p<.001**

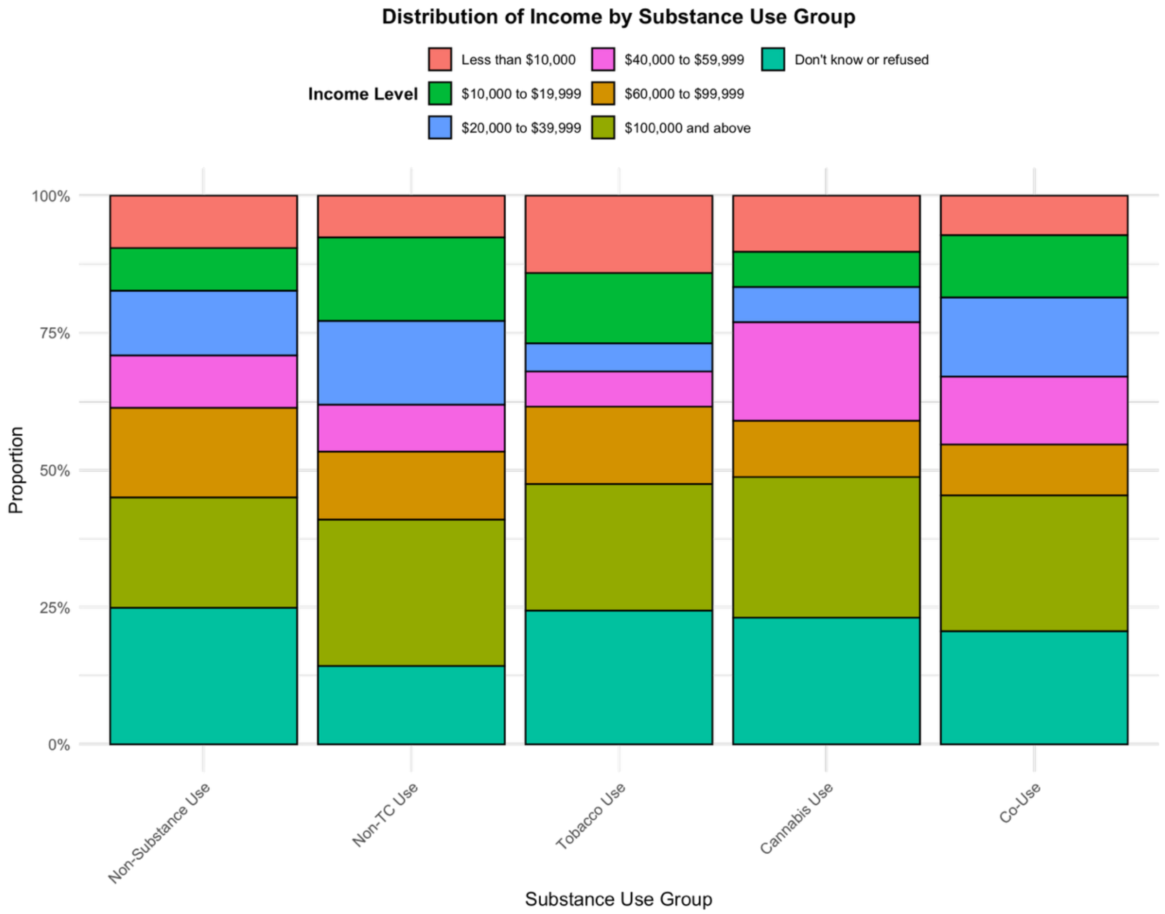

Supplemental Figure 1. Socioeconomic Status Does Not Differ Across CHR Substance Use Groups. Socioeconomic status was measured using household income across CHR substance use groups (n=730). Group differences were assessed using a Kruskal-Wallis test (two-sided). There were no significant differences in socioeconomic status between groups (H(4)=4.67, p=.32).

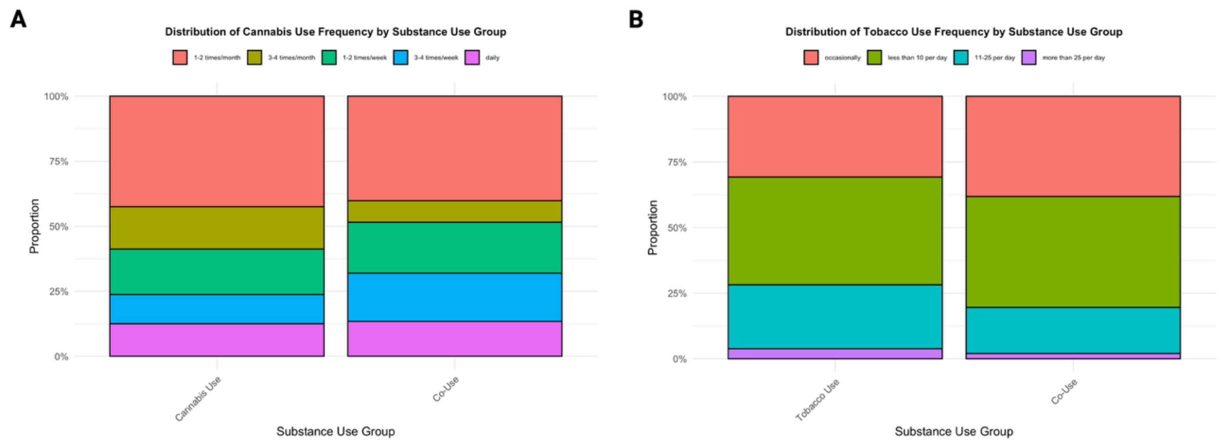

Supplemental Figure 2. Cannabis and Tobacco Use Frequency Does Not Differ Between Cannabis- or Tobacco Users and Co-Users. Cannabis use frequency as measured by the AUS/DUS did not differ between Cannabis Users and Co-Users (Kruskal-Wallis, two-sided, H(1)=0.77, p=.38, n=176, 2A). Tobacco use frequency, also measured by the AUS/DUS, did not differ between Tobacco Users and Co-Users (Kruskal-Wallis, two-sided, H(1)=1.92, p=.17, n=175, 2B).

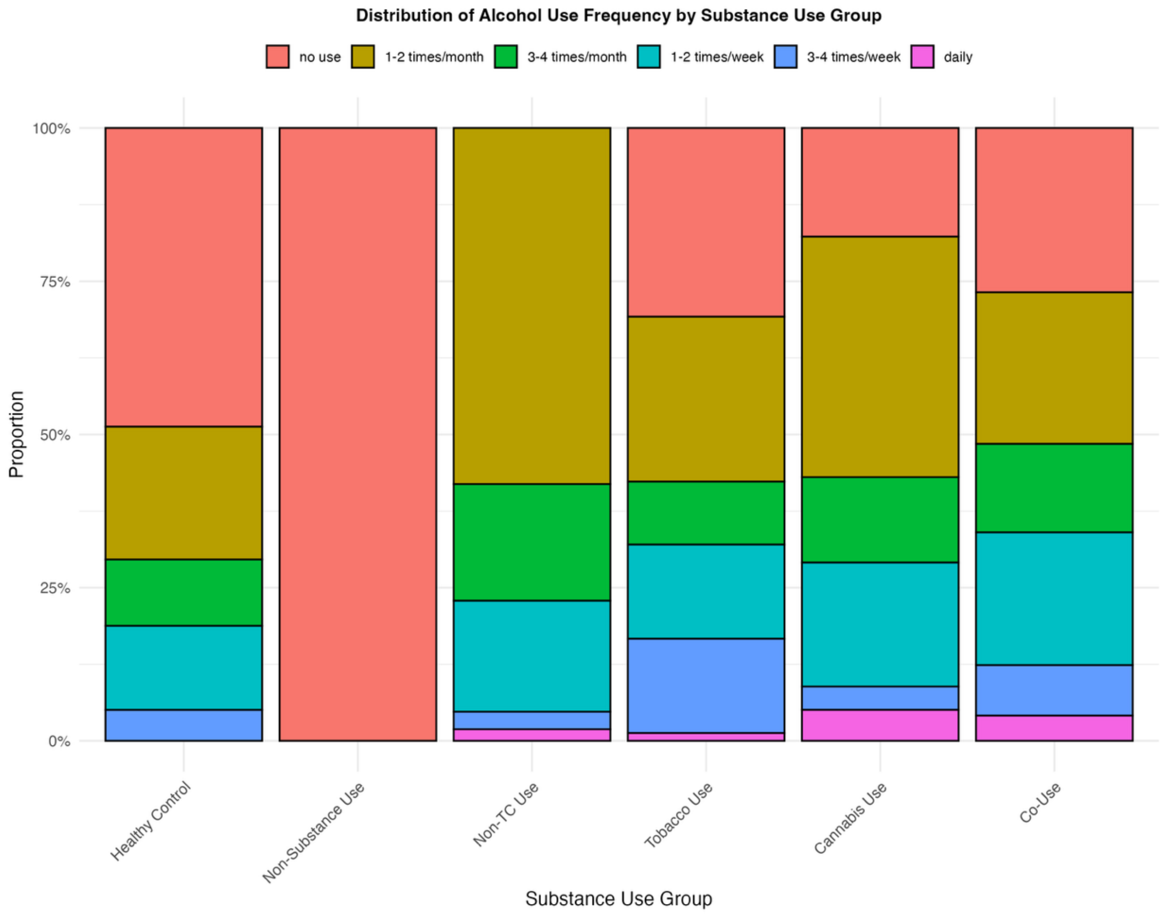

Supplemental Figure 3. Alcohol Use Frequency Does Not Differ Among Tobacco Users, Cannabis Users, and Co-Users in the Sample. Alcohol use frequency did not differ among Tobacco Users, Cannabis Users, and Co-Users in the CHR group (Kruskal-Wallis, two-sided, $H(2)=0.38$, $p=.83$, $n=254$).

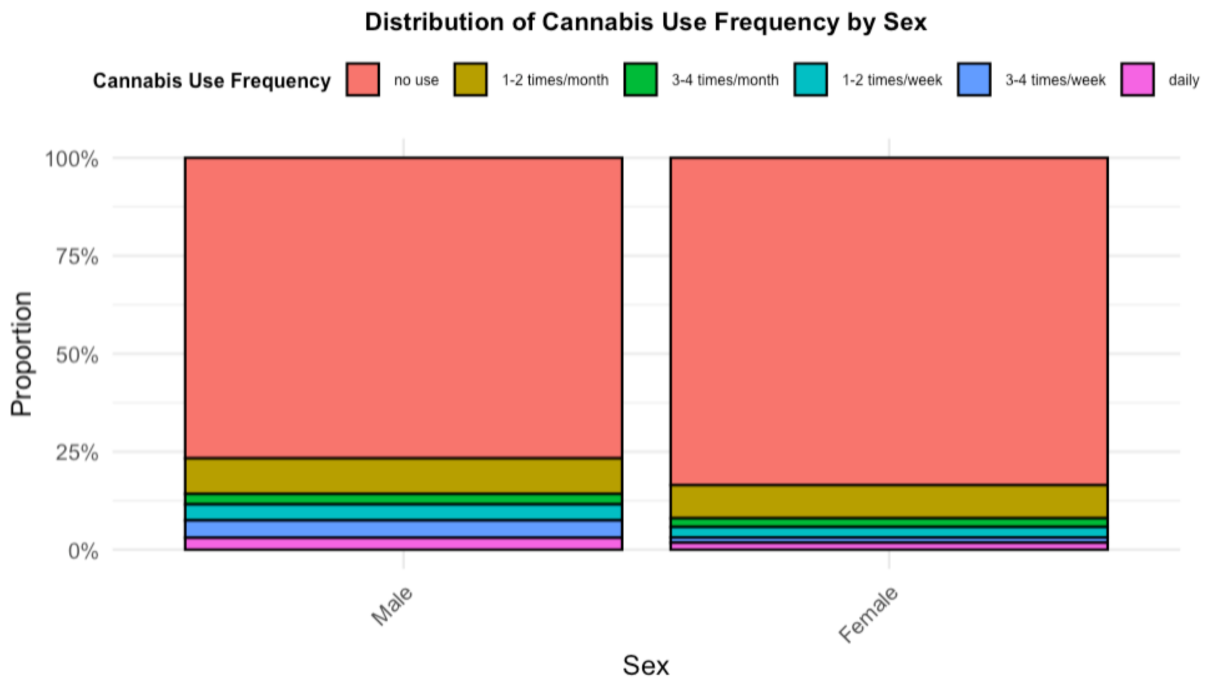

Supplemental Figure 4. Males Have Higher Cannabis Use Frequency Than Females. Across the combined sample (CHR and Healthy Controls), males used cannabis more frequently than females (Kruskal-Wallis, two-sided, H(1)=8.41, p=.004, n=1,012), as measured by the AUS/DUS.

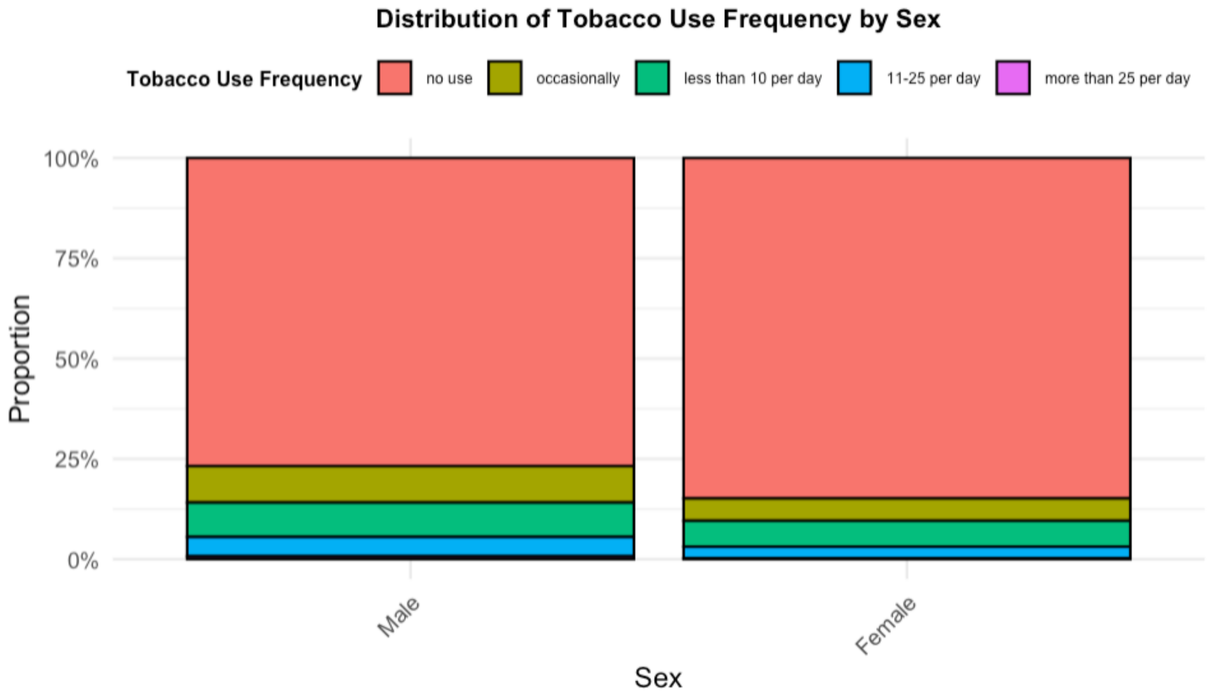

Supplemental Figure 5. Males Have Higher Tobacco Use Frequency Than Females. Across the combined sample (CHR and Healthy Controls), males used tobacco more frequently than females (Kruskal-Wallis, two-sided, $H(1)=10.17$, $df=1$, $p=.001$, $n=1{,}012$), as measured by the AUS/DUS.

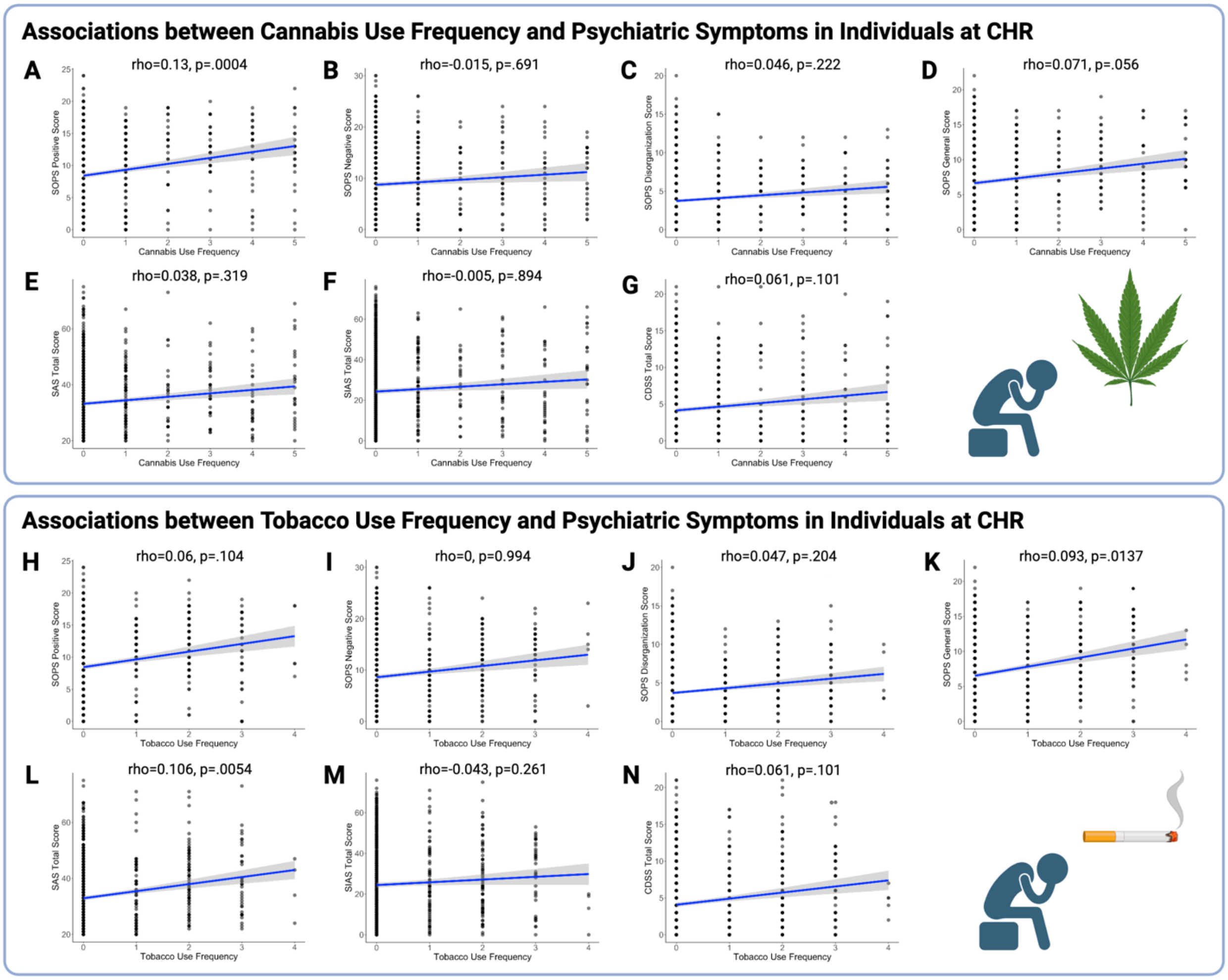

Supplemental Figure 6. In the CHR Group, More Frequent Cannabis Use is Associated with Higher Positive Symptom Severity, while More Frequent Tobacco use is Associated with Higher Anxiety. In the CHR population alone (n=734), more frequent cannabis use was associated with greater positive symptoms (Spearman ρ = 0.13, p=.0004, A). However, more frequent cannabis use was not associated with negative (Spearman ρ = -0.015, p=.69, B), disorganization (Spearman ρ = 0.046, p=.22, C), or general psychosis symptoms (Spearman ρ = 0.071, p=.056, D), or anxiety (Spearman ρ = 0.038, p=.32, E), social anxiety (Spearman ρ = -0.005, p=.89, F), or depression (Spearman ρ = 0.061, p=.10, G). Higher tobacco use frequency was associated with higher anxiety severity (Spearman ρ = 0.106, p=.0054, L). Tobacco use frequency was not associated with positive (Spearman ρ = 0.06, p=.10, H), negative (Spearman ρ = 0, p=.99, I), disorganization (Spearman ρ = 0.047, p=.20, J), or general psychosis symptoms (Spearman ρ = 0.093, p=.014, K), or social anxiety (Spearman ρ = -0.043, p=.26, M), or depression severity

(Spearman ρ = .061, p=.10, N). Bonferroni-corrected threshold p=.007. All tests are two-sided spearman correlations. Shaded bands represent 95% confidence intervals around the linear regression line.

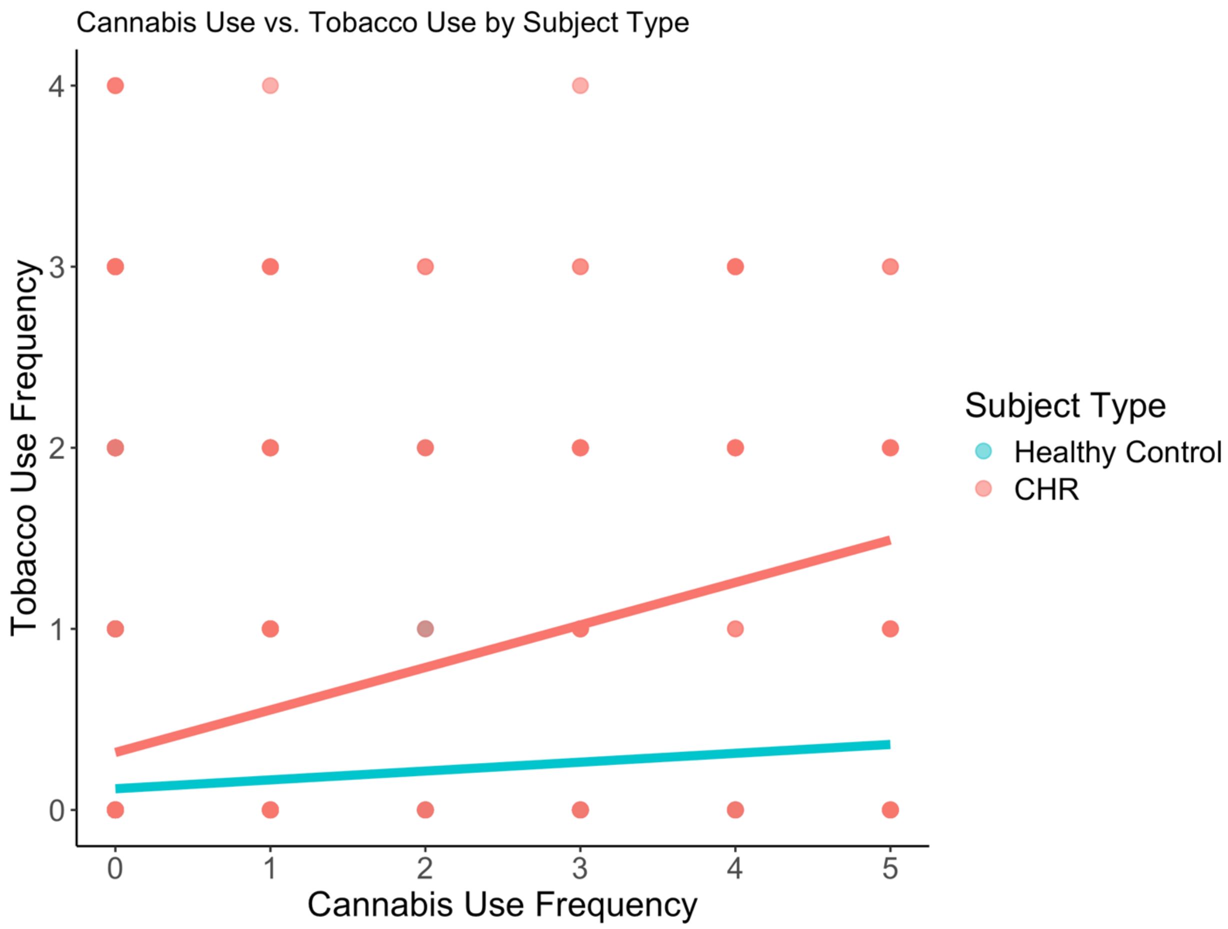

Supplemental Figure 7. More Frequent Cannabis Use is Associated with More Frequent Tobacco Use. In a linear regression model to predict tobacco use frequency controlling for age, sex, site, diagnosis, cannabis frequency, and the diagnosis*cannabis use frequency interaction ($F(12,996)=15.97$, $p<.001$, $n=1,012$), older age ($Estimate=0.0262$, $SE=0.0058$, $t=4.51$, $p<0.001$), CHR diagnosis ($Estimate=0.224$, $SE=0.058$, $t=3.866$, $p<.001$), male sex ($Estimate=-0.109$, $SE=0.0489$, $t=-2.225$, $p=0.026$), and the diagnosis*cannabis use frequency interaction ($Estimate=0.157$, $SE=0.0654$, $t=2.404$, $p=.016$) were predictors of tobacco use such that CHR participants with more frequent cannabis use also had more frequent tobacco use. The Atlanta, New York, North Carolina, and Calgary sites predicted greater tobacco use ($p<.05$).

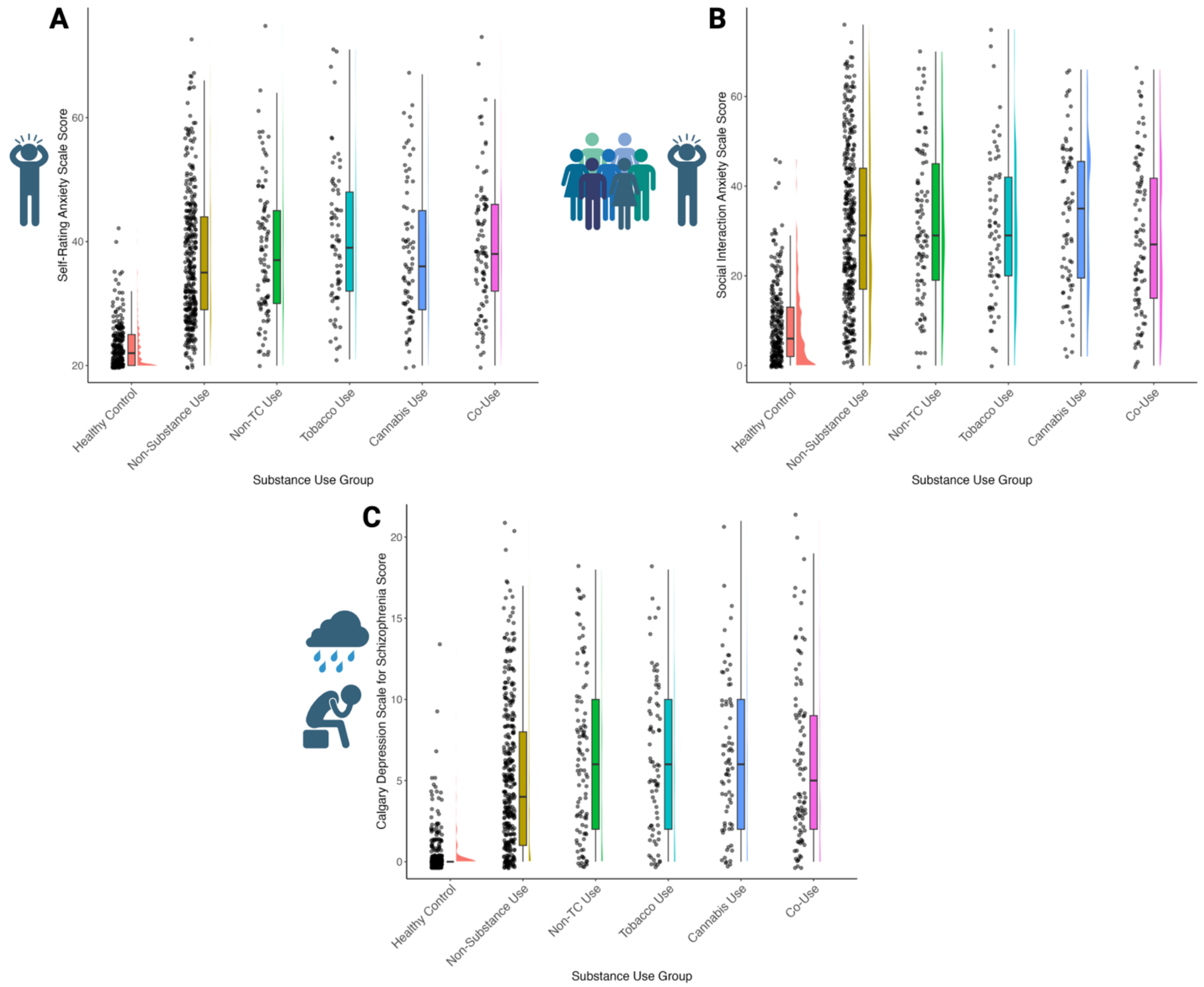

Supplemental Figure 8. Anxiety and Depression Severity Do Not Differ Across CHR Substance Use Groups. Anxiety (SAS, A), social anxiety (SIAS, B), and depression (CDSS, C) scores did not differ across CHR substance use groups (one-way ANOVA, two-sided, Bonferroni-corrected threshold p=.007; SAS: F(4,683)=2.11, p=.08, n=688; SIAS: F(4,679)=0.90, p=.46, n=684; CDSS: F(4,708)=2.88, p=.02, n=713). Healthy Controls had significantly lower anxiety, social anxiety, and depression scores than all other groups (SAS: F(5,942)=97.81, p<.001, n=948; SIAS: F(5,936)=75.44, p<.001, n=942; CDSS: F(5,982)=68.78, p<.001, n=988) but their significance bars have been omitted for simplicity. Boxplots display the median (center line), 25th and 75th percentiles (box bounds), and 1.5×IQR (whiskers); individual data points are shown as dots.

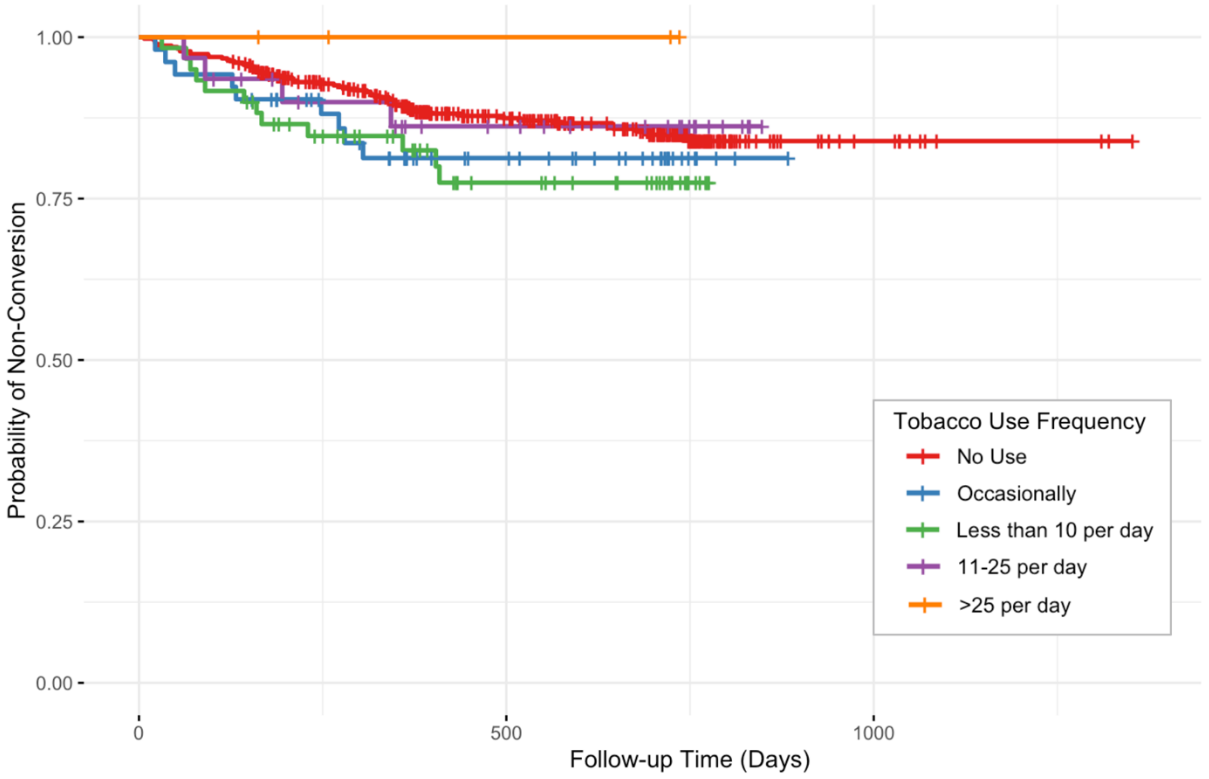

Supplemental Figure 9. Tobacco Use Frequency is Not Associated with Higher Conversion Risk.

Tobacco use frequency at baseline was not significantly associated with higher risk of conversion to psychosis (*HR* = 1.12, 95% CI [0.90–1.40], *p* = .32). Neither age nor sex were associated with conversion (*HRage* = 0.97, 95% CI [0.92–1.03], *p* = 0.290; *HRsex* = 0.76, 95% CI [0.48–1.19], *p* = 0.227). Kaplan-Meier survival curves for time to conversion to psychosis by baseline ordinal tobacco use. The curve is plotted for the purpose of descriptive survival patterns and was not adjusted for age or sex. Cox proportional hazards model, two-sided, controlling for age and sex. N=734 CHR participants.

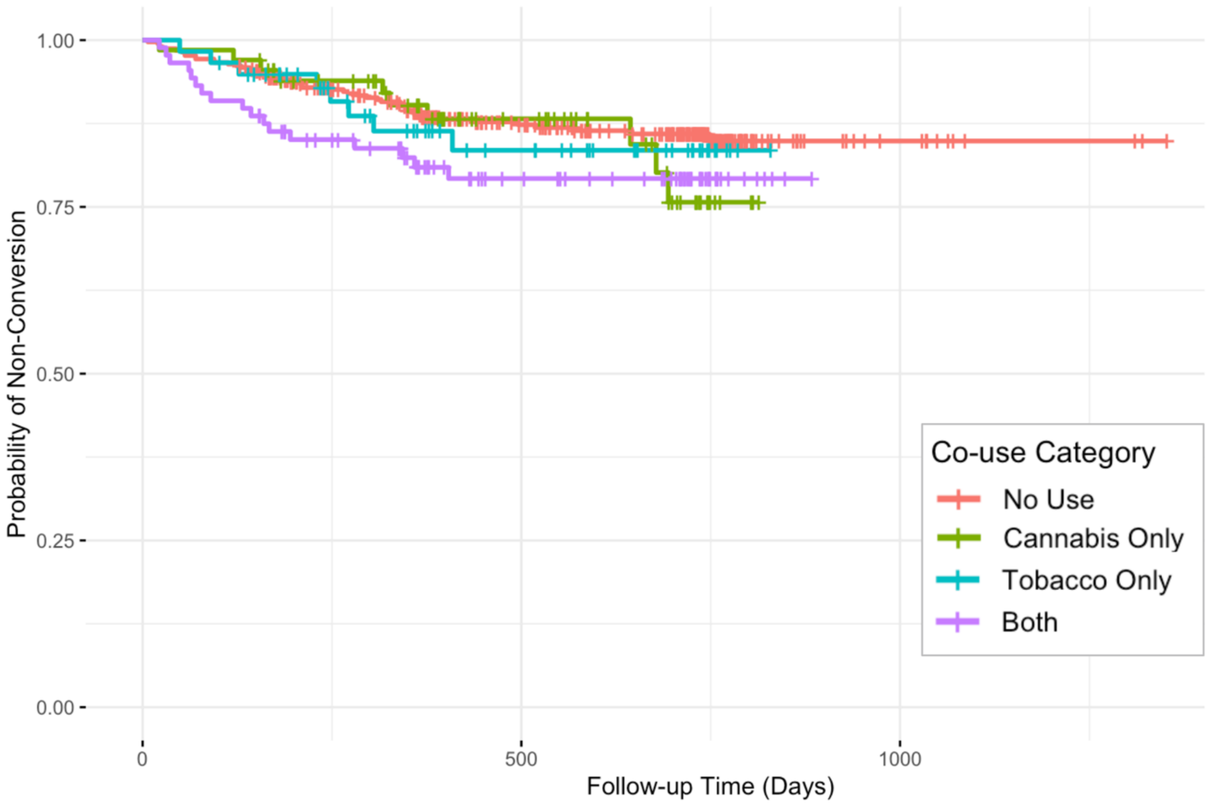

Supplemental Figure 10. Categorical Tobacco and Cannabis Co-Use is Not Associated with Higher Risk of Conversion to Psychosis. When we defined tobacco and cannabis use categorically (no use, tobacco use only, cannabis use only, co-use), co-use of cannabis and tobacco was associated with higher risk of conversion compared to no use of either substance (*HR* = 1.69, 95% CI [0.96–2.97], *p* = .070), but did not reach significance. Cannabis use only (*HR* = 1.29, 95% CI [0.64–2.57], *p* = .47) and tobacco use only (*HR* = 1.25, 95% CI [0.58–2.67], *p* = .57) did not significantly predict conversion compared to no use of either substance. Neither age nor sex were associated with conversion (*HRage* = 0.97, 95% CI [0.91–1.02], *p* = 0.238; *HRsex* = 0.78, 95% CI [0.49–1.22], *p* = 0.274). Kaplan-Meier survival curves for time to conversion to psychosis by categorical substance use group. The curve is plotted for the purpose of descriptive survival patterns and was not adjusted for age or sex. Cox proportional hazards model, two-sided, controlling for age and sex. N=734 CHR participants (480 no tobacco or cannabis use, 78 tobacco use only, 79 cannabis use only, 97 co-use).

References

1. McGlashan T, Walsh B, Woods S. *The Psychosis Risk Syndrome: Handbook For Diagnosis and Follow-Up*. 1st ed. Oxford University Press; 2010.

2. Miller TJ, McGlashan TH, Rosen JL, et al. Prodromal assessment with the structured interview for prodromal syndromes and the scale of prodromal symptoms: predictive validity, interrater reliability, and training to reliability. *Schizophr Bull*. 2003;29(4):703-715. doi:10.1093/oxfordjournals.schbul.a007040

3. Drake R, Mueser K, McHugo G. Clinician rating scales: Alcohol use scale (AUS), drug use scale (DUS), and substance abuse treatment scale (SATS). In: *Outcome Assessment in Clinical Practice*. First. Williams and Wilkins; 1996:113-116.

4. Ward HB, Lawson MT, Addington J, et al. Tobacco use and psychosis risk in persons at clinical high risk. *Early Intervention in Psychiatry*. 2019;13(5):1173-1181. doi:10.1111/eip.12751

5. Kendler KS, Lönn SL, Sundquist J, Sundquist K. Smoking and Schizophrenia in Population Cohorts of Swedish Women and Men: A Prospective Co-Relative Control Study. *Am J Psychiatry*. 2015;172(11):1092-1100. doi:10.1176/appi.ajp.2015.15010126

6. Zung WWK. A Rating Instrument For Anxiety Disorders. *Psychosomatics*. 1971;12(6):371-379. doi:10.1016/S0033-3182(71)71479-0

7. Mattick RP, Clarke JC. Development and validation of measures of social phobia scrutiny fear and social interaction anxiety. *Behav Res Ther*. 1998;36(4):455-470. doi:10.1016/s0005-7967(97)10031-6

8. Addington D, Addington J, Maticka-Tyndale E. Assessing depression in schizophrenia: the Calgary Depression Scale. *Br J Psychiatry Suppl*. 1993;(22):39-44.
