# Supplementary material for: Cannabis and tobacco co-use predicts psychosis in clinical high risk cohorts
Source: Nat Ment Health. 2026 May 12;4(6):941–50. doi: 10.1038/s44220-026-00648-y (PMC13259916; doi:10.1038/s44220-026-00648-y)
Supplement: Supplementary file 2 — Reporting Summary [file 44220_2026_648_MOESM2_ESM.pdf]

nature portfolio

Corresponding author(s): Heather Ward

Last updated by author(s): Apr 8, 2026

Reporting Summary

Nature Portfolio wishes to improve the reproducibility of the work that we publish. This form provides structure for consistency and transparency in reporting. For further information on Nature Portfolio policies, see our Editorial Policies and the Editorial Policy Checklist.

Statistics

For all statistical analyses, confirm that the following items are present in the figure legend, table legend, main text, or Methods section.

n/a	Confirmed	
☐	☒	The exact sample size (*n*) for each experimental group/condition, given as a discrete number and unit of measurement
☐	☒	A statement on whether measurements were taken from distinct samples or whether the same sample was measured repeatedly
☐	☒	The statistical test(s) used AND whether they are one- or two-sided<br>*Only common tests should be described solely by name; describe more complex techniques in the Methods section.*
☐	☒	A description of all covariates tested
☐	☒	A description of any assumptions or corrections, such as tests of normality and adjustment for multiple comparisons
☐	☒	A full description of the statistical parameters including central tendency (e.g. means) or other basic estimates (e.g. regression coefficient) AND variation (e.g. standard deviation) or associated estimates of uncertainty (e.g. confidence intervals)
☐	☒	For null hypothesis testing, the test statistic (e.g. F, t, r) with confidence intervals, effect sizes, degrees of freedom and P value noted<br>*Give P values as exact values whenever suitable.*
☒	☐	For Bayesian analysis, information on the choice of priors and Markov chain Monte Carlo settings
☒	☐	For hierarchical and complex designs, identification of the appropriate level for tests and full reporting of outcomes
☐	☒	Estimates of effect sizes (e.g. Cohen's *d*, Pearson's *r*), indicating how they were calculated

Our web collection on statistics for biologists contains articles on many of the points above.

Software and code

Policy information about availability of computer code

Data collection	No software was used for data collection.
Data analysis	ll analyses were conducted in RStudio (Version 2023.03.1+446) using alpha<.05, except as indicated in results to correct for multiple comparisons. All analyses were conducted using R version 4.5.1 (2025-06-13) and the following packages: survival and survminer.

For manuscripts utilizing custom algorithms or software that are central to the research but not yet described in published literature, software must be made available to editors and reviewers. We strongly encourage code deposition in a community repository (e.g. GitHub). See the Nature Portfolio guidelines for submitting code & software for further information.

Data

Policy information about availability of data

All manuscripts must include a data availability statement. This statement should provide the following information, where applicable:

- Accession codes, unique identifiers, or web links for publicly available datasets
- A description of any restrictions on data availability
- For clinical datasets or third party data, please ensure that the statement adheres to our policy

Data are publicly available on the National Institute of Mental Health Data Archive.

Research involving human participants, their data, or biological material

Policy information about studies with human participants or human data. See also policy information about sex, gender (identity/presentation), and sexual orientation and race, ethnicity and racism.

Reporting on sex and gender	Sex was determined by self-report
Reporting on race, ethnicity, or other socially relevant groupings	Race/ethnicity was self-reported
Population characteristics	age, sex, site, diagnosis were included as covariates
Recruitment	data was collected as part of the North American Prodrome Longitudinal Study-2
Ethics oversight	Prior to participation, all participants provided written informed consent (or, if under age 18, informed assent with parental consent) in accordance with the institutional review boards of Beth Israel Deaconess Medical Center, Boston, Massachusetts; Emory University, Atlanta, Georgia; University of Calgary, Alberta, Canada; University of California, Los Angeles; University of California, San Diego; The University of North Carolina at Chapel Hill; Yale University, New Haven, Connecticut; and Zucker Hillside Hospital, New York.

Note that full information on the approval of the study protocol must also be provided in the manuscript.

Field-specific reporting

Please select the one below that is the best fit for your research. If you are not sure, read the appropriate sections before making your selection.

☐ Life sciences ☒ Behavioural & social sciences ☐ Ecological, evolutionary & environmental sciences

For a reference copy of the document with all sections, see nature.com/documents/nr-reporting-summary-flat.pdf

Behavioural & social sciences study design

All studies must disclose on these points even when the disclosure is negative.

Study description	NAPLS2 is a longitudinal case-control study studying individuals at CHR for psychosis across 8 sites in North America.
Research sample	NAPLS2 is a longitudinal case-control study studying individuals at CHR for psychosis across 8 sites in North America. Individuals meeting CHR criteria (N=734) and healthy controls (N=280) were enrolled.
Sampling strategy	No further sampling needed.
Data collection	Participants underwent assessment at baseline and every 6 months for two years and upon conversion to psychosis (if applicable) from January 2009 to April 2013.
Timing	As above
Data exclusions	Only baseline assessments were used for this analysis.
Non-participation	All baseline data was included
Randomization	There was no randomization

Reporting for specific materials, systems and methods

We require information from authors about some types of materials, experimental systems and methods used in many studies. Here, indicate whether each material, system or method listed is relevant to your study. If you are not sure if a list item applies to your research, read the appropriate section before selecting a response.

Materials & experimental systems

n/a	Involved in the study
☒	☐ Antibodies
☒	☐ Eukaryotic cell lines
☒	☐ Palaeontology and archaeology
☒	☐ Animals and other organisms
☒	☐ Clinical data
☒	☐ Dual use research of concern
☒	☐ Plants

Methods

n/a	Involved in the study
☒	☐ ChIP-seq
☒	☐ Flow cytometry
☒	☐ MRI-based neuroimaging

Plants

Seed stocks	N/A
Novel plant genotypes	N/A
Authentication	N/A
